# Reprogramming immunosuppressive myeloid cells facilitates immunotherapy for colorectal cancer

**DOI:** 10.15252/emmm.202012798

**Published:** 2020-12-07

**Authors:** Weiqiang Lu, Weiwei Yu, Jiacheng He, Wenjuan Liu, Junjie Yang, Xianhua Lin, Yuanjin Zhang, Xin Wang, Wenhao Jiang, Jian Luo, Qiansen Zhang, Huaiyu Yang, Shihong Peng, Zhengfang Yi, Shancheng Ren, Jing Chen, Stefan Siwko, Ruth Nussinov, Feixiong Cheng, Hankun Zhang, Mingyao Liu

**Affiliations:** ^1^ Shanghai Key Laboratory of Regulatory Biology Institute of Biomedical Sciences and School of Life Sciences East China Normal University Shanghai China; ^2^ Department of Urology Changhai Hospital Second Military University Shanghai China; ^3^ School of Basic Medical Sciences Ningxia Medical University Yinchuan China; ^4^ Department of Molecular and Cellular Medicine Institute of Biosciences and Technology Texas A&M University Health Science Center Houston TX USA; ^5^ Cancer and Inflammation Program Leidos Biomedical Research, Inc., Frederick National Laboratory for Cancer Research Sponsored by the National Cancer Institute Frederick MD USA; ^6^ Department of Human Molecular Genetics and Biochemistry Sackler School of Medicine Tel Aviv University Tel Aviv Israel; ^7^ Genomic Medicine Institute, Lerner Research Institute Cleveland Clinic Cleveland OH USA; ^8^ Department of Molecular Medicine Cleveland Clinic Lerner College of Medicine Case Western Reserve University Cleveland OH USA; ^9^ Case Comprehensive Cancer Center Case Western Reserve University School of Medicine Cleveland OH USA

**Keywords:** colorectal cancer, immunosuppressive myeloid cells, immunotherapy, prostaglandin E2 receptor 4, Cancer, Immunology

## Abstract

Immune checkpoint blockade (ICB) has a limited effect on colorectal cancer, underlining the requirement of co‐targeting the complementary mechanisms. Here, we identified prostaglandin E2 (PGE_2_) receptor 4 (EP4) as the master regulator of immunosuppressive myeloid cells (IMCs), which are the major driver of resistance to ICB therapy. PGE_2_‐bound EP4 promotes the differentiation of immunosuppressive M2 macrophages and myeloid‐derived suppressor cells (MDSCs) and reduces the expansion of immunostimulated M1 macrophages. To explore the immunotherapeutic role of EP4 signaling, we developed a novel and selective EP4 antagonist TP‐16. TP‐16 effectively blocked the function of IMCs and enhanced cytotoxic T‐cell‐mediated tumor elimination *in vivo*. Cell co‐culture experiments revealed that TP‐16 promoted T‐cell proliferation, which was impaired by tumor‐derived CD11b^+^ myeloid cells. Notably, TP‐16 and anti‐PD‐1 combination therapy significantly impeded tumor progression and prolonged mice survival. We further demonstrated that TP‐16 increased responsiveness to anti‐PD‐1 therapy in an IMC‐related spontaneous colorectal cancer mouse model. In summary, this study demonstrates that inhibition of EP4‐expressing IMCs may offer a potential strategy for enhancing the efficacy of immunotherapy for colorectal cancer.

## Graphical abstract

Immunosuppressive myeloid cells (IMCs) are a prominent driver of immunotherapy resistance in colorectal cancer. This study identifies EP4 as a master regulator of IMCs and highlights blockade of EP4 as a novel therapeutic strategy for enhancing immunotherapy in colorectal cancer.

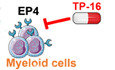

**The paper explained**

**Problem**

Immune checkpoint blockade (ICB) has emerged as the standard therapy in patients with refractory cancers owing to its unprecedented and durable responses. However, ICB activity is lost in colorectal cancer, especially in the microsatellite‐stable population. Increasing evidence has indicated that immunosuppressive myeloid cells (IMCs) are a prominent driver of immunotherapy resistance in colorectal cancer. Given the plasticity and complexity of IMCs in the tumor microenvironment, novel strategies are required to target these tumor‐associated myeloid cells and to enhance cancer treatment efficacy.

**Results**

We identified prostaglandin E2 receptor 4 (EP4) as the master regulator of PGE_2_ in IMCs. PGE_2_‐bound EP4 promoted the differentiation of immunosuppressive M2 macrophages and myeloid‐derived suppressor cells (MDSCs) and reduced the expansion of immunostimulated M1 macrophages. Treatment with TP‐16, a novel EP4 antagonist, blocked the function of IMCs (M2 macrophages and MDSCs) and enhanced cytotoxic T‐cell‐mediated colorectal cancer elimination *in vivo*. Cell co‐culture experiments revealed that TP‐16 promoted the proliferation of T cells, which was impaired by tumor‐derived CD11b + myeloid cells. Notably, the combination therapy of TP‐16 and anti‐PD‐1 antibody significantly hampered tumor progression and prolonged survival in syngeneic mouse models. Finally, TP‐16 increased responsiveness to anti‐PD‐1 therapy in an azoxymethane/dextran sodium sulfate (AOM/DSS) model, an IMC‐related colorectal cancer mouse model.

**Impact**

These observations suggest that PGE_2_‐bound EP4 promotes the immunosuppressive activity of IMCs. Chemical inhibition of EP4 by TP‐16 induces a functional switch in myeloid cells from immunosuppression to immunostimulation and enhanced cytotoxic T‐cell activation. TP‐16 acts synergistically with anti‐PD‐1 therapy in colorectal cancer mouse models, offering a potential approach for improving the efficacy of checkpoint‐based immunotherapies in colorectal cancer patients.

## Introduction

Colorectal cancer has a high incidence and mortality rate (Siegel *et al*, 2019). In 2018, more than 1.8 million new cases and 881,000 deaths were reported worldwide (Bray *et al*, 2018). Approximately 20% of patients have distant metastatic disease at initial diagnosis, and half of the patients develop metastases during disease progression (Chiappa *et al*, 2009). The prognosis for patients with distant‐stage disease is dismal, with a 5‐year relative survival rate of only 14% (Siegel *et al*, 2020). Therefore, the development of innovative treatments is an urgent requirement to improve the clinical benefit for patients with advanced or metastatic colorectal cancer.

Immune checkpoint blockade (ICB) has emerged as the standard therapy for many cancers due to its unprecedented and durable responses in patients with refractory cancers (Hoos, 2016; Ganesh *et al*, 2019). For instance, anti‐CTLA4 antibody was approved for metastatic melanoma, and anti‐PD‐(L)1 antibodies were approved for a wide range of cancer types, such as melanoma, lung cancer, and renal carcinoma (Topalian *et al*, 2015; Patel & Minn, 2018). The potential benefits of ICB have been reported in approximately 15% of colorectal cancer patients with defective mismatch repair (microsatellite instability–high, MSI‐H). However, the activity is lost in the microsatellite‐stable (MSS) population, representing the majority of colorectal cancer patients (Pitt *et al*, 2016; Overman *et al*, 2018).

Increasing evidence has indicated that immunosuppressive myeloid cells (IMCs) are a prominent driver of immunotherapy resistance in colorectal cancer (Le *et al*, 2017; Liao *et al*, 2019). IMCs mainly comprise tumor‐associated macrophages (TAMs) and myeloid‐derived suppressor cells (MDSCs) (Gabrilovich *et al*, 2012). TAMs accumulation is associated with poor prognosis and is a potential diagnostic biomarker for treatment stratification (Steidl *et al*, 2010; Lu‐Emerson *et al*, 2013). A high frequency of MDSCs is generally associated with poor disease progression and therapy resistance in colorectal cancer (Solito *et al*, 2011; Marvel & Gabrilovich, 2015; Kumar *et al*, 2016). Thus, abrogating IMC‐mediated adaptive immune response deficiency would offer a potential strategy for tumor immunotherapy.

Prostaglandin E2 (PGE_2_), a bioactive lipid with pro‐tumor activity, acts through several mechanisms in colorectal cancer (Obermajer & Kalinski, 2012; Luan *et al*, 2015; Martinez‐Colon & Moore, 2018). IMCs have been reported as the dominant target cells affected by PGE_2_ through cognate G‐protein coupled receptors (GPCRs), such as E‐type prostanoid receptors 1‐4 (EP1‐4) (Wu *et al*, 2019). Among these receptors, EP4 is highly expressed in IMCs and plays an important role in the differentiation of TAMs and MDSCs in the tumor microenvironment (Sinha *et al*, 2007; Sugimoto & Narumiya, 2007). However, the immunotherapeutic effect of targeting EP4‐expressing IMCs in colorectal cancer remains elusive.

In this study, we found that PGE_2_‐bound EP4 induces a functional switch in myeloid cells from immunostimulation to immunosuppression. Chemical inhibition of EP4 by the new EP4 antagonist, TP‐16, significantly reprograms IMCs and enhances cytotoxic T‐cell activation. TP‐16 acts synergistically with anti‐PD‐1 therapy in colorectal cancer mouse models, offering a potential approach for improving the efficacy of checkpoint‐based immunotherapies.

## Results

### EP4 is the master regulator of IMCs

**Figure 1:**
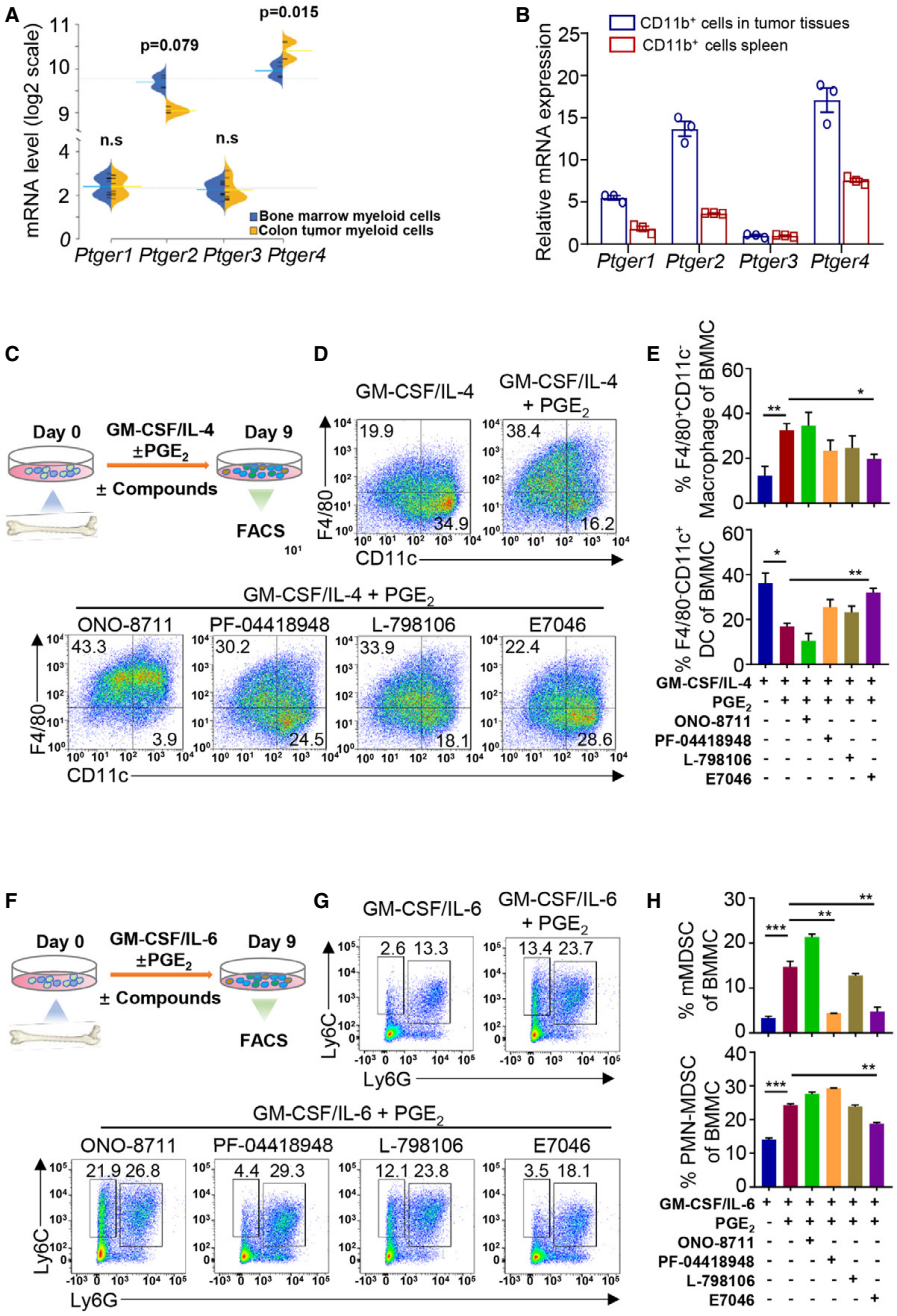
EP4 is a master regulator of IMCs The expression of EP subtypes in bone marrow myeloid cells and colon tumor myeloid cells. The short black lines indicate individual expressions, and the blue and yellow lines indicate the average expression level in each condition. The gray dotted lines represent the overall average between Ptger1 and Ptger3 or Ptger2 and Ptger4.The expression of EP subtypes in CD11b^+^ cells isolated from tumor tissues and spleen of CT26 tumor‐bearing mice by magnetic bead separation (*n* = 3).Schematic diagram of the granulocyte–macrophage colony‐stimulating factor (GM‐CSF)/interleukin (IL)‐4‐induced mouse monocyte DCs/macrophages differentiation assay. Cells were treated with specific antagonists of distinct EP receptors: ONO‐8711, EP1; PF‐04418948, EP2; L‐798106, EP3; and E7046, EP4.Representative flow cytometry plots revealing the effects of prostaglandin E2 (PGE_2_) and EP1‐4 antagonists on mouse bone marrow monocyte dendritic cell (DC)/macrophage differentiation (*n* = 3).The frequencies of F4/80^+^CD11C^−^ macrophages and F4/80^−^CD11C^+^ DCs under varying treatments as analyzed by flow cytometry analysis (*n* = 3).Schematic representation of the GM‐CSF/IL‐6‐induced mouse bone marrow monocyte myeloid‐derived suppressor cell (MDSC) differentiation assay (*n* = 3).Representative flow cytometry plots revealing the effects of PGE_2_ and EP1‐4 antagonists on mouse bone marrow‐derived‐MDSC differentiation. Upper subpanel: vehicle group and PGE_2_ group. Lower subpanel: ONO‐8711 (EP1 antagonist) group, PF‐04418948 (EP2 antagonist) group, L‐798106 (EP3 antagonist) group, and E7046 (EP4 antagonist) group. (*n* = 3).The frequencies of Ly6C^+^Ly6G^−^ mMDSC and Ly6C^mid^Ly6G^+^ PMN‐MDSCs under varied treatments as analyzed by flow cytometry analysis (*n* = 3). Data information: Data are presented as mean ± SEM. (A) Kolmogorov–Smirnov test; (E, H) two‐tailed unpaired Student’s *t*‐test was performed; *P < 0.05; **P < 0.01; ***P < 0.001. Exact *P* values and statistical tests are listed in Appendix Table S8.

EP1‐4 constitutes a subfamily of cell surface receptors of the immunosuppressive molecule, PGE_2_. We performed bioinformatic analyses and found that in the myeloid cells of primary bone marrow (BM), the expression of EP2 and EP4 was abundant, whereas the expression of EP1 and EP3 was minimal (Fig 1A). Among EP1‐4, EP4 was significantly up‐regulated (*P* = 0.015) in colon tumor myeloid cells compared to BM myeloid cells (Fig 1A) (Yang *et al*, 2011). Accordingly, in a CT26 tumor‐bearing mouse model, the expression level of EP4 was higher than those of EP1–3 in CD11b^+^ myeloid cells isolated from tumor tissues and the spleen (Fig 1B).

Further, we examined the role of distinct EP subtypes by using specific antagonists in myeloid cell differentiation. Isolated mouse BM cells were stimulated with granulocyte–macrophage colony‐stimulating factor (GM‐CSF) and interleukin‐4 (IL‐4) in the presence or absence of PGE_2_ *in vitro* (Fig 1C). Dendritic cells (DCs, F4/80^–^CD11c^+^) had a greater proportion of GM‐CSF/IL‐4 differentiated myeloid cells than macrophages (F4/80^+^CD11c^–^), whereas PGE_2_ treatment largely suppressed DC differentiation, and correspondingly promoted macrophage differentiation (Fig 1D and E). Notably, we found that chemical inhibition of EP4 effectively reduced macrophage differentiation and rescued DC differentiation in the presence of PGE_2_ (Fig 1D and E). Further, we differentiated mouse BM cells into MDSCs *in vitro* by treating them with GM‐CSF and IL‐6 (Fig 1F). The exposure of mouse BM cells to GM‐CSF/IL‐6 led to the generation of immature MDSCs expressing Ly6C^+^Ly6G^–^ or Ly6C^mid^Ly6G^+^ (Fig 1G and H). Remarkably, PGE_2_ enhanced the differentiation and expansion of MDSCs (Fig 1G and H). Intriguingly, EP1 and EP3 antagonists had little effect on MDSC and the EP2 blockade was able to reduce the differentiation of monocytic MDSC (mMDSCs, Ly6C^+^Ly6G^–^CD11b^+^) but not polymorphonuclear MDSC (PMN‐MDSCs, Ly6C^mid^Ly6G^+^CD11b^+^), which is consistent with previous studies (Shi *et al*, 2014; Rodriguez‐Ubreva *et al*, 2017). Importantly, chemical inhibition of EP4 decreased the expansion of both mMDSCs and PMN‐MDSCs. In summary, EP4 was the main receptor of PGE_2_ in boosting the differentiation and expansion of immunosuppressive macrophages and MDSCs.

### TP‐16 is a novel, selective EP4 antagonist

**Figure 2:**
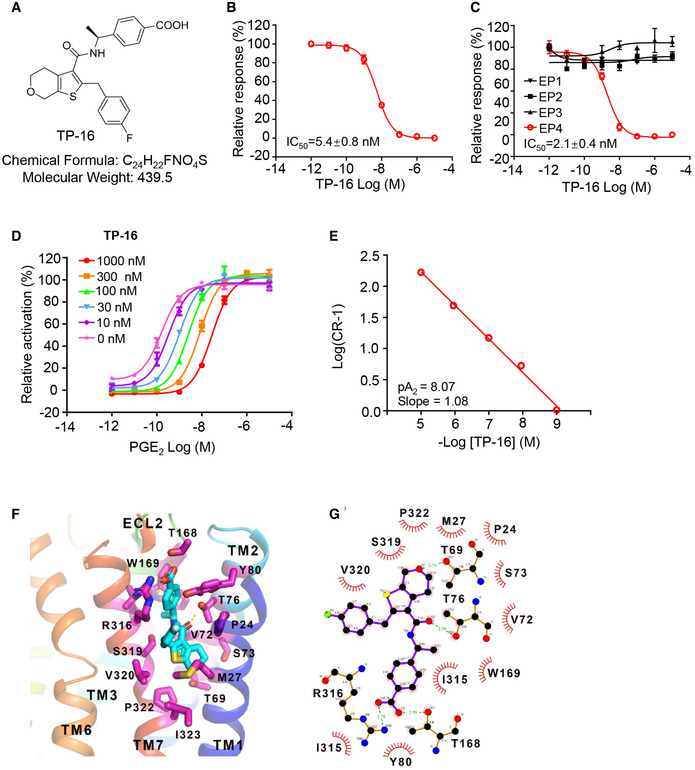
Discovery and characterization of a potent and selective EP4 antagonist, TP‐16 Chemical structure of TP‐16.Dose–effect curve of TP‐16 in GloSensor™ cAMP assay in EP4‐ expressing 293 cells (*n* = 3).Dose–effect curves of TP‐16 in PGE_2_‐induced calcium flux assay (*n* = 3).The Schild plot of PGE_2_ in the presence of varying concentrations of TP‐16. TP‐16 shifted the dose–response curve of PGE_2_‐induced intracellular cAMP levels in a dose‐dependent manner (*n* = 3).The pA_2_ value and slope of the Schild plot.Docked pose of TP‐16 with critical residues in the putative binding pocket of human EP4 protein. EP4 is shown as a color cartoon, the residues important for the interaction are depicted in magenta sticks, and TP‐16 is shown as a cyan stick figure.The LIGPLOT diagram summarizes key interactions between TP‐16 (purple lines) and residues that originate from EP4. T69, T76, T168, and R316 establish hydrogen bonds with TP‐16. Semicircles with radiating lines indicate non‐polar interactions. Data information: Data are presented as mean ± SEM from three independent experiments with similar results.

**Figure EV1:**
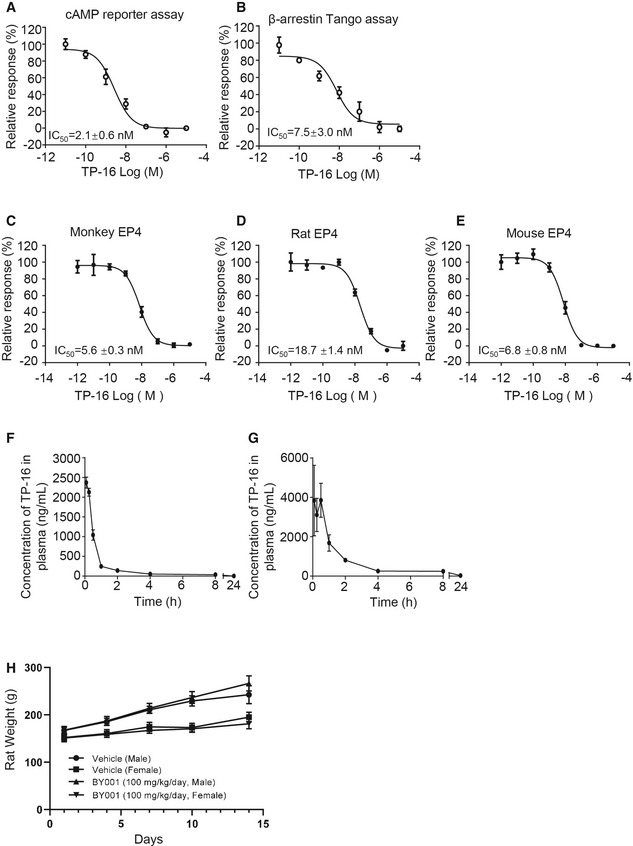
Antagonistic activity, pharmacokinetic profiles, and safety of TP‐16 ADose–response of TP‐16 in cAMP reporter gene assay in HEK293 cells. Data are presented as mean ± SEM derived from three independent experiments (*n* = 3).BDose–effect curves of TP‐16 in PGE_2_‐induced β‐arrestin Tango assay. Data are presented as mean ± SEM derived from three independent experiments (*n* = 3).C–EThe antagonistic activities of TP‐16 on EP4 were determined by a calcium flux assay by overexpressing Gα16 protein in CHO cells. Dose–response curves of TP‐16 against EP4: monkey (C), rat (D), and mouse (E). Data are presented as mean ± SEM derived from three independent experiments (*n* = 3).F, GThe plasma concentration over time profiles of TP‐16 after intravenous and oral administration of TP‐16 at a dose of 1 and 10 mg/kg, respectively. Results are presented as mean ± SEM of three CD1 mice (*n* = 3).HBody weights of male and female SD rats orally administered with TP‐16 (100 mg/kg/day) in 14‐day repeat‐dose. Data are presented as means ± SD (*n* = 3/sex/group). Data information: Data are presented as mean ± SEM except for H.

We then sought to explore novel EP4 antagonists with improved drug‐likeness because of the unfavorable pharmacokinetics properties of current clinical compounds (i.e., grapiprant) (Markovic *et al*, 2017). TP‐16 is a new thienopyran‐containing small molecule derived from an extensive medicinal chemistry campaign (see Appendix Methods). TP‐16 demonstrated potent EP4 antagonistic activity in HEK293‐EP4 cells with a half minimal inhibitory concentration (IC_50_) value of 2.1 ± 0.6 nM in the cAMP‐responsive element (CRE) luciferase assay (Figs 2A and EV1A). A strong antagonistic activity of TP‐16 was observed in the GloSensor™ cAMP assay with an IC_50_ value of 5.4 ± 0.8 nM (Fig 2B). EP4 recruits β‐arrestin, and the Tango is a validated assay for evaluating ligand‐induced GPCR/β‐arrestin 2 interaction (Kroeze *et al*, 2015). The Tango assay revealed that TP‐16 dose‐dependently blocked PGE_2_‐induced EP4/β‐arrestin interaction in HEK293 cells with an IC_50_ value of 7.5 ± 3.0 nM (Fig EV1B). EP4 was reported to trigger calcium flux in CHO‐Gα16 cells (Wu *et al*, 2018). The calcium flux assay revealed that TP‐16 was a potent EP4 antagonist in human (Fig 2C), monkey (Fig EV1C), rat (Fig EV1D), and mouse (Fig EV1E) with IC_50_ values of 2.1 ± 0.4 nM, 5.6 ± 0.3 nM, 18.7 ± 1.4 nM, and 6.8 ± 0.8 nM, respectively. In addition, this assay revealed that TP‐16 had a > 3000‐fold higher selectivity for human EP4 than human EP1 ‐3 (IC_50_> 10 µM for EP1, EP2, and EP3) (Fig 2C). Moreover, the LANCE Ultra cAMP assay and calcium flux assay revealed that other 25 GPCRs were not affected by TP‐16 (IC_50_ > 10 μM) (Appendix Tables S1 and S2), indicating high target selectivity.

PGE_2_ increased the intracellular cAMP level in a dose‐dependent manner in HEK293‐EP4 cells (Fig 2D). An increase in the concentration of TP‐16 induced a rightward shift in PGE_2_ concentration–response curves without changing the maximal cAMP accumulation. Subsequently, schild plot analysis yielded a pA2 value of 8.07 with a slope of 1.08 (Fig 2E).

To investigate the detailed interaction between EP4 and TP‐16, in silico molecular docking was performed using AutoDock (Morris *et al*, 2009). The crystal structure of human EP4 bound to ONO‐AE3‐208 was retrieved from the Protein Data Bank (PDB ID: 5YWY) (Toyoda *et al*, 2019). The docking grid was created based on the ONO‐AE3‐208 binding pocket. The lowest energy docking model was selected for subsequent interaction analysis. We found that the predicted binding pocket of TP‐16 was mainly composed of transmembrane (TM) 1, TM2, TM3, TM7, and extracellular loop 2 (ECL2) (Fig 2F and G). Specifically, the carboxyl group of TP‐16 interacted with the guanidinium group of Arg316 and the hydroxyl group of Thr168 through a salt bridge. This interaction mode is similar to the antagonist, ONO‐AE3‐208, binding in the crystal structure of the EP4 complex (Toyoda *et al.*, 2019). In addition, the amide group could form a hydrogen bond with the hydroxyl group of Thr76, and the oxygen atom in the six‐element ring of TP‐16 interacted with Thr69.

### Ideal pharmacokinetics and safety of TP‐16

We then evaluated the pharmacokinetic properties of TP‐16 in CD1 mice. The mean plasma concentration–time curves are presented in Fig EV1F and G, and the key pharmacokinetic parameters are shown in Appendix Table S3. The Cmax value of a single dose of TP‐16 (10 mg/kg, p.o.) at 0.5 h was 3851.8 ng/ml. The elimination half‐life (t1/2) was 5.4 h, and the AUC_0‐24_ was 8399.8 h*ng/ml. i.v. administration of TP‐16 (1 mg/kg) had an AUC0‐24 of 2137.6 h*ng/ml. Calculating the ratio of oral AUC_0‐∞_ to intravenous AUC_0‐∞_ indicated that TP‐16 had a favorable oral bioavailability of 40.1%. In addition, we assessed the metabolic stability of TP‐16 in human and mouse liver microsome. The T_1/2_ values of TP‐16 were> 581 and 235 min, and the hepatic intrinsic clearance values of TP‐16 were < 4.7 and 39.3 ml/min/kg in humans and mice, respectively (Appendix Table S4).

To evaluate the safety of TP‐16, we performed a 14‐day toxicity study in rats. Male (*n* = 3) and female (*n* = 3) Sprague Dawley (SD) rats were orally administered TP‐16 (100 mg/kg, daily) for 14 days, and the body weight, blood parameters, and organ morphology were recorded. Our results demonstrated that TP‐16 was well tolerated without statistically significant weight loss during the 14‐day treatment (Fig EV1H). Hematological analysis revealed that no adverse changes occurred in rats of either sex (Appendix Table S5). Moreover, no abnormalities of organ morphology (Appendix Table S6) were observed. In summary, TP‐16 exhibited good pharmacokinetic and safety profiles.

### TP‐16 suppresses tumor growth in syngeneic murine tumor models

**Figure 3:**
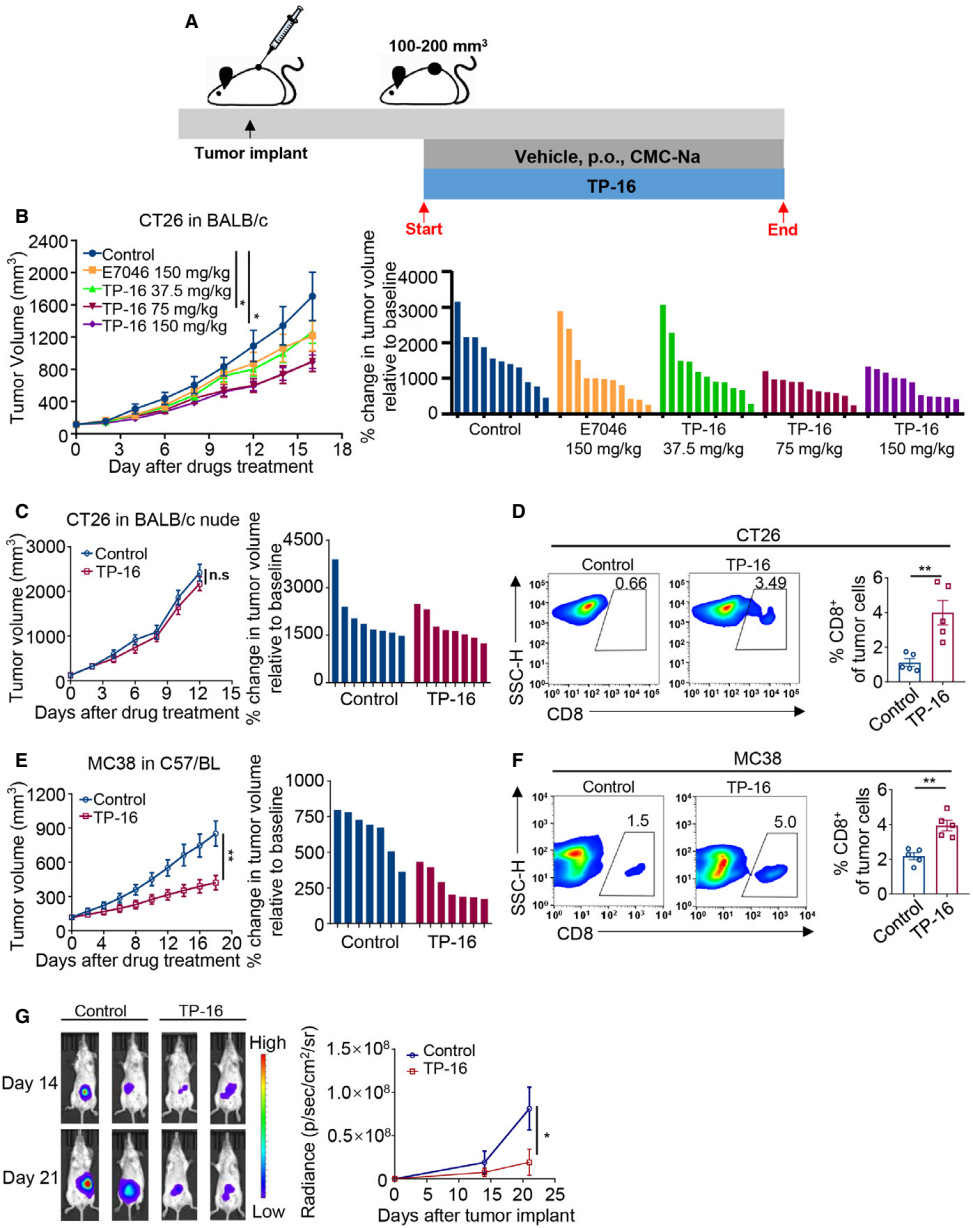
EP4 antagonist TP‐16 robustly suppresses the tumor growth in murine syngeneic tumor models Schematic illustration of the establishment of the murine syngeneic tumor models and drug treatment schedule. Established tumor models were orally treated daily with vehicle or TP‐16 when tumor volumes reached 100‐200 mm^3^.The anti‐tumor activities of E7046 (150 mg/kg) and TP‐16 (37.5, 75, and 150 mg/kg) in CT26 tumor‐bearing BALB/c mice (*n* = 11 per group). Tumor growth curves in the CT26 model (left panel). Bar graphs show the individual changes in tumor growth relative to after and before drug administration (right panel). Tumor volumes reached 100–200 mm^3^ on day 7.Representative graph showing that the anti‐tumor activity of TP‐16 is lost in CT26 tumors engrafted into BALB/c nude mice (left panel) (*n* = 8). Bar graphs show the individual changes in tumor growth relative to after and before drug administration (right panel). Tumor volumes reached 100–200 mm^3^ on day 7.Single‐cell suspensions of tumor tissues from CT26 tumor‐bearing BALB/c mice treated with vehicle or TP‐16 for 2 weeks were analyzed for immune cell infiltration by flow cytometry analysis. Representative flow cytometry and dot plots of intratumoral frequencies of CD8^+^ T cells (*n* = 5).Growth curves (left panel) and relative change after 18 days treatment (right panel) in colon cancer (MC38) xenograft tumors in C57BL/6 mice (*n* = 7). Tumor volumes reached 100–200 mm^3^ on day 7.Single‐cell suspensions of tumor tissues from MC38 tumor‐bearing C57BL/6 mice treated with vehicle or TP‐16 for 2 weeks were analyzed for immune cell infiltration by flow cytometry analysis. Representative flow cytometry and dot plots of intratumoral frequencies of CD8^+^ T cells (*n* = 5).The representative bioluminescence images (left panel) and quantitative growth curves (right panel) of an orthotopic colorectal cancer mouse model, CT26‐Luc model (*n* = 4). Data information: Data are presented as mean ± SEM. (B) One‐way analysis of variance (ANOVA), Tukey's multiple comparison test; *P < 0.05. (C‐G) two‐tailed unpaired Student’s *t‐*test was performed; *P < 0.05; **P < 0.01. Exact *P* values and statistical tests are listed in Appendix Table S8. Source data are available online for this figure.

**Figure EV2:**
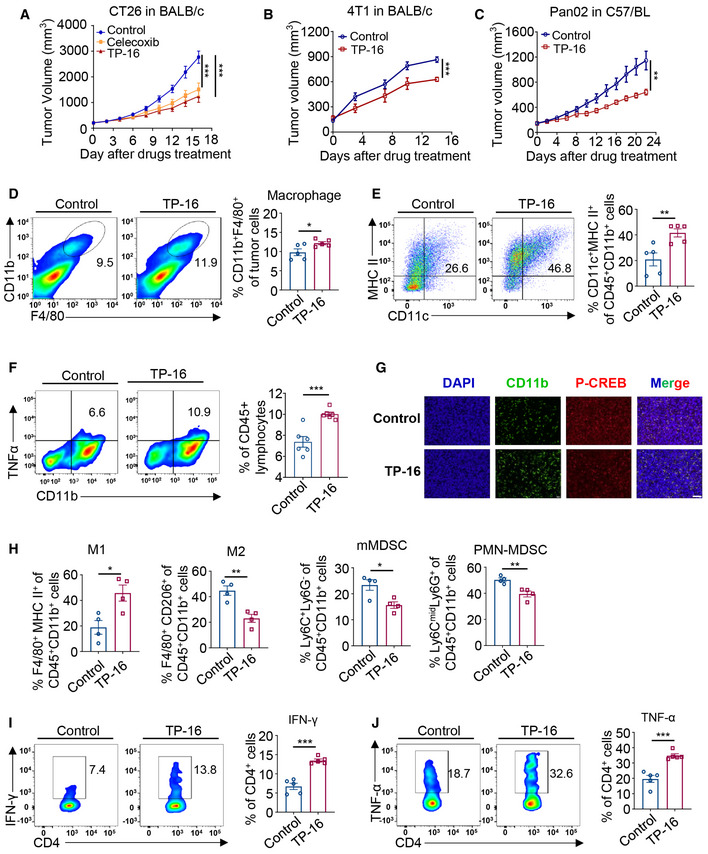
Effect of EP4 inhibition by TP‐16 on tumor growth and subsets of intratumoral immune cells AThe growth curves of CT26 xenograft tumors in BALB/c mice. CT26 tumor‐bearing mice were treated with TP‐16 (75 mg/kg, p.o., daily) or celecoxib (100 mg/kg, p.o., daily) for 16 days when tumor volumes reached 100‐200 mm^3^ (day 7). Data are presented as mean ± SEM. One‐way analysis of variance (ANOVA), Tukey's multiple comparison; ***P < 0.001. *n* = 6.B, CThe anti‐tumor activity of TP‐16 on 4T1 xenograft tumors in BALB/c mice and Pan02 xenograft tumors in C57BL/6 mice. Mice were treated with TP‐16 (75 mg/kg, po, daily) when tumor volumes reached 100–200 mm^3^ (day 7). Data are presented as mean ± SEM. A two‐tailed unpaired Student’s *t*‐test was performed; **P < 0.01; ***P < 0.001 (*n* = 8).D, ESingle‐cell suspensions of tumors from CT26‐bearing BALB/c mice treated with vehicle or 75 mg/kg TP‐16 for 2 weeks were analyzed for intratumoral immune cells by flow cytometry analysis. Representative graphs and quantification of CD11b^+^F4/80^+^ macrophages (D) and CD45^+^CD11b^+^CD11c^+^MHC II^+^ dendritic cells (DCs) (E). Data are presented as mean ± SEM. A two‐tailed unpaired Student’s *t*‐test was performed; *P < 0.05; **P < 0.01 (*n* = 5).FRepresentative graphs and quantification of TNF‐α in CD11b^+^ cells isolated from the CT26 xenograft tumors (*n* = 6). Data are presented as mean ± SEM. A two‐tailed unpaired Student’s *t*‐test was performed, ***P < 0.001 versus the vehicle control group.GRepresentative immunofluorescence images of p‐CREB in CD11b^+^ cells of CT26 tumor tissue. Scale bars, 50 μm.HThe changes of myeloid cell compartment in an orthotopic colorectal cancer mouse model, CT26‐Luc model (*n* = 4). A two‐tailed unpaired Student’s *t‐*test was performed, *P < 0.05, **P < 0.01 versus vehicle control group.I, JRepresentative graphs and quantification of CD4 + T‐cell activation markers IFN‐γ (H) and TNF‐α (I) (*n* = 5). Data are presented as mean ± SEM derived from three independent experiments. A two‐tailed unpaired Student’s t‐test was performed, ***P < 0.001 versus the vehicle control group. Source data are available online for this figure.

To assess the *in vivo* anti‐tumor potential of TP‐16, we used syngeneic tumor models. We evaluated the effects of different doses of TP‐16 (37.5, 75, and 150 mg/kg) on colorectal cancer cell growth in CT26 mouse bearing BALB/c mice. Animals were orally administered with TP‐16 or control vehicle (0.5% carboxymethylcellulose sodium in phosphate‐buffered saline (PBS)) after the tumor volume reached 100‐200 mm^3^ (Fig 3A). TP‐16 treatment resulted in statistically significant tumor growth inhibition (TGI) at 75 mg/kg (%TGI = 47.4%) and 150 mg/kg (%TGI = 47.6%) and modest inhibition at 37.5 mg/kg (%TGI = 26.2%) over a period of 16 days. Notably, TP‐16 showed greater efficacy than E7046, a selective EP4 antagonist in phase I trials (Albu *et al*, 2017), at the same dosage (150 mg/kg, Fig 3B). TP‐16 (75 mg/kg) was superior to the COX‐2 inhibitor celecoxib (100 mg/kg) in the CT26 model (Fig EV2A). In addition, we evaluated TP‐16 in CT26‐ tumor‐bearing BALB/c nude mice. The efficacy of TP‐16 was lost after BALB/c nude mice bearing CT26 tumors were treated with 75 mg/kg TP‐16, indicating that functional T cells were required for TP‐16 anti‐tumor activity (Fig 3C). Flow cytometry (FACS) analyses further confirmed that TP‐16 induced an increased infiltration of CD8^+^ T cell into CT26 tumors (Fig 3D). Of note, even 100 µM TP‐16 did not affect the viability of mouse cancer cells (CT26, MC38, 4T1, and Panc02), human cancer cells (HCT116, HCT8, HT29, and DLD1) or endothelial cells (HUVEC) *in vitro* (Appendix Fig S1).

Thereafter, we investigated the *in vivo* efficacy of TP‐16 in an MC38 colorectal cancer model. Daily oral administration of TP‐16 (75 mg/kg) significantly impaired tumor growth (%TGI = 50.6) (Fig 3E). Moreover, CD8^+^ leukocyte accumulation was observed in MC38 colon cancer model after TP‐16 treatment (Fig 3F), which further indicated immune‐mediated anti‐tumor efficacy. Intriguingly, the anti‐cancer effects of TP‐16 were observed in breast cancer 4T1 (%TGI = 27.3%) (Fig EV2B) and pancreatic cancer Pan02 (%TGI = 44.0%) (Fig EV2C), suggesting a common underlying mechanism in these tumors.

We further evaluated the potency of TP‐16 using an orthotopic, syngeneic colorectal cancer mouse model. Luciferase‐labeled CT26 (CT26‐Luc) cells were injected into the mouse cecum wall, and orthotopic tumor growth was monitored using an IVIS spectrum imaging system via an intraperitoneal injection of luciferin. Tumors in the control vehicle group rapidly grew and spread in the abdominal area (Fig 3G). In line with the results obtained in the subcutaneous tumor models, TP‐16 treatment triggered tumor regression in the CT26‐Luc orthotopic model with a %TGI of 76.22%. In addition, no significant change was observed in the body weight of these mice, suggesting that TP‐16 treatment was well tolerated in mice at the given doses (Appendix Fig S2).

### TP‐16 reprograms IMCs and enhances anti‐tumor immunity

**Figure 4:**
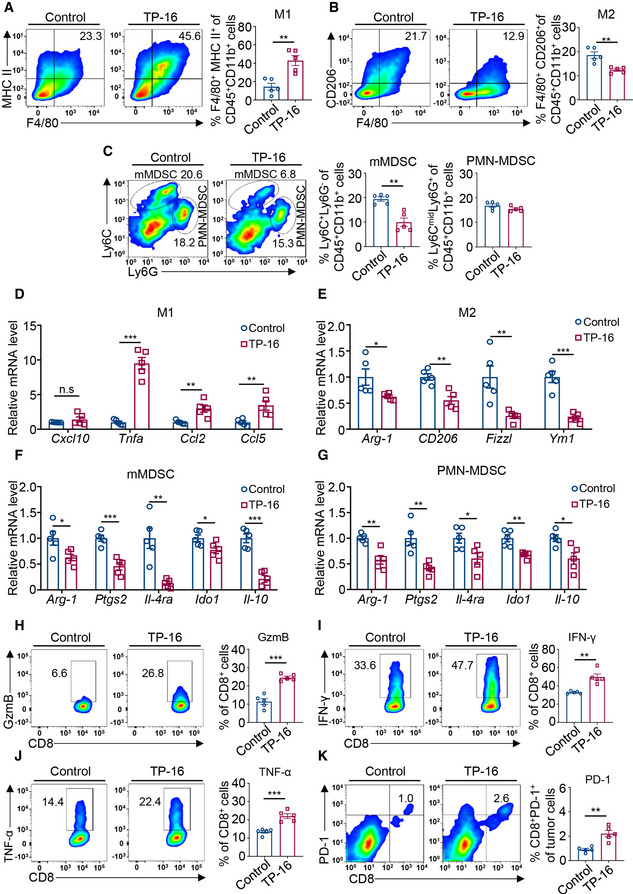
EP4 antagonist TP‐16 reprograms tumor‐associated myeloid cells (IMCs) and enhances anti‐tumor immunity A, BTumors from CT26 tumor‐bearing BALB/c mice treated with vehicle or TP‐16 for 2 weeks were harvested, and tumor single‐cell suspensions were analyzed for tumor‐associated myeloid cells by flow cytometry analysis. Representative graphs and quantification of F4/80^+^MHC‐II^+^ immunosuppressive myeloid cells (IMCs)‐M1 macrophages (A) and F4/80^+^CD206^+^ IMCs‐M2 macrophages (B) gated on CD45^+^CD11b^+^ myeloid cells (*n* = 5).CRepresentative flow cytometry analysis and quantification of Ly6C^+^Ly6G^‐^ IMC‐monocytic myeloid‐derived suppressor cell (mMDSC) and Ly6C^mid^Ly6G^+^ IMC‐PMN‐MDSC frequencies gated on CD45^+^CD11b^+^ myeloid cells (*n* = 5).D–GRelative expression of selected M1, M2, and MDSC markers on flow‐sorted M1 macrophages (D), M2 macrophages (E), mMDSCs (F), and PMN‐MDSCs (G) from CT26 tumors. Data are normalized to β‐actin expression levels (*n* = 5).H–KRepresentative flow analysis and quantification of CD8^+^ T‐cell activation markers: Granzyme B (H), IFN‐γ (I), TNF‐α (J), and PD‐1 (K) (*n* = 5). Data information: Data are presented as mean ± SEM. (A‐K) Two‐tailed unpaired Student’s *t*‐test; *P < 0.05; **P < 0.01; ***P < 0.001. Exact *P* values and statistical tests are listed in Appendix Table S8.

We investigated the effects of TP‐16 on IMCs composition and their immunosuppressive function in the tumor microenvironment. The total proportions of macrophages (CD11b^+^F4/80^+^) and DC (CD45^+^CD11b^+^MHCII^+^CD11c^+^) were increased in TP‐16 treated CT26 tumors, compared with the control vehicle group (Figs EV2D and 2E). Particularly, TP‐16 treatment switched the polarization of macrophages from the CD45^+^CD11b^+^F4/80^+^CD206^+^ immunosuppressive M2 phenotype (pro‐tumor) to the CD45^+^CD11b^+^F4/80^+^MHC‐II^+^ proinflammatory M1 phenotype (anti‐tumor) (Fig 4A and B). Meanwhile, the proportion of Ly6C^+^Ly6G^–^CD45^+^CD11b^+^ monocytic cells (mMDSCs) was significantly decreased following TP‐16 treatment, though no significant difference was observed in the proportion of Ly6C^mid^Ly6G^+^CD45^+^CD11b^+^ granulocytic cells (PMN‐MDSCs) (Fig 4C).

Thereafter, we examined the expression levels of inflammatory and immune‐related genes in IMCs derived from tumor tissues. M1 macrophages, M2 macrophages, mMDSCs, and PMN‐MDSCs were sorted from CT26 tumors treated with control vehicle or TP‐16 by FACS analysis. Compared with the control vehicle group, mRNA expression of inflammatory cytokines (*Tnfa, Ccl2*, and *Ccl5*) was increased in M1‐like cells, whereas that of classical M2 markers (*Arg‐1, CD206, Fizzl*, and *Ym1*) was reduced in M2‐like cells of TP‐16‐treated group (Fig 4D and E). Consistently, increased protein expression of TNF‐α was also observed in isolated CD11b^+^ myeloid cells (Fig EV2F). Furthermore, the expression of MDSC markers (for both mMDSC and PMN‐MDSC), such as *Arg‐1, Ptgs2, IL‐4ra, Ido1*, and *Il‐10,* was decreased in the TP‐16‐treated group (Fig 4F and G). Consistent with the aforementioned results, we observed that TP‐16 treatment significantly decreased the levels of p‐CREB, a biomarker of PGE_2_‐EP4 signaling, in CD11b^+^ cells of CT26 tumors (Fig EV2G). Of note, the changes observed in the myeloid cell compartment were found in an orthotopic colorectal cancer mouse model, the CT26‐Luc model (Fig EV2H). Overall, EP4 inhibition by TP‐16 could reverse the suppressive function of myeloid cells in the tumor microenvironment.

We next asked whether a less immunosuppressive phenotype of myeloid cells induced by TP‐16 was accompanied by enhanced CD8^+^ cytotoxic T‐cell activation in CT26 tumors. As mentioned previously, TP‐16 treatment significantly increased the infiltration of CD8^+^ T cells into tumors (Fig 3D). We further found that CD8^+^ T cells isolated from TP‐16‐treated CT26 tumors expressed high levels of effector enzyme GzmB and effector factors (IFN‐γ, and TNF‐α), compared with those isolated from control vehicle‐treated tumors (Fig 4H–J). The expression of PD‐1, a marker of activated cytotoxic T cells, was higher in CD8^+^ T cells after TP‐16 treatment (Fig 4K). Similarly, the expression levels of IFN‐γ (Fig EV2I) and TNF‐α (Fig EV2J) were higher in CD4^+^ T cells. Overall, TP‐16 reprogrammed IMCs toward a less immunosuppressive state and enhanced T‐cell‐mediated anti‐tumor immunity *in vivo*.

### TP‐16 reverses the immunosuppressive capacity of IMCs

**Figure EV3:**
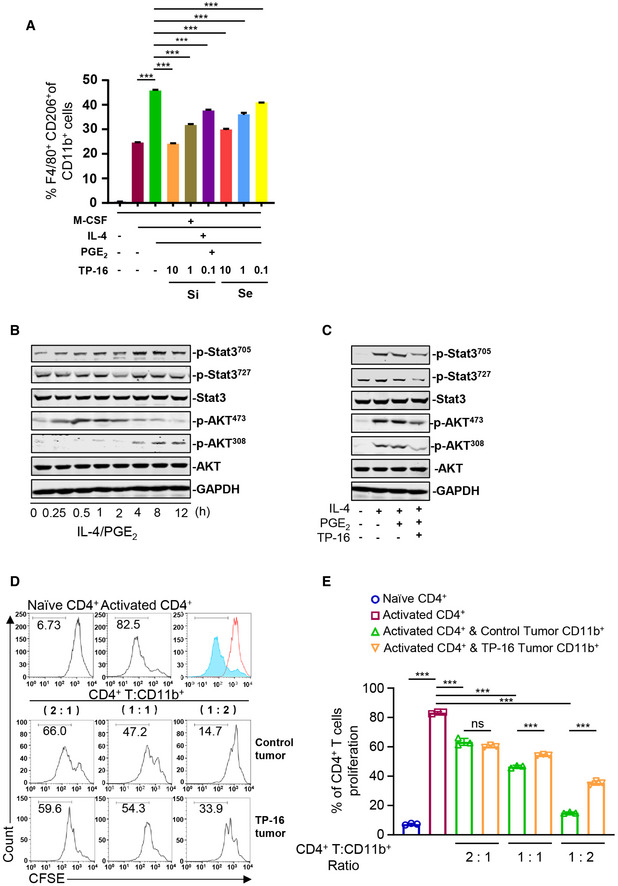
TP‐16 suppresses M2 macrophage differentiation and reverses T‐cell proliferation when cultured with tumor‐infiltrating CD11b^+^ myeloid cells AMouse bone marrow cells were cultured with M‐CSF/IL‐4/PGE_2_ for 6 days and varied concentrations of TP‐16 was simultaneously (day 0) or sequentially (day 3) added. The generation of M2 macrophages (F4/80^+^CD206^+^) was analyzed by flow cytometry analysis. Si: simultaneous TP‐16 treatment; Se: sequential TP‐16 treatment. Data are presented as mean ± SEM. One‐way analysis of variance (ANOVA) and Tukey's multiple comparison test were performed; ***P < 0.001 (*n* = 3).B, CMouse bone marrow‐derived macrophages (BMMs) were stimulated with 20 ng/ml mouse recombinant IL‐4 alone or in combination with 100 nM PGE_2_, in the absence or presence of 10 μM TP‐16 for the indicated time. The expression of p‐STAT3^705^, p‐STAT3^727^, p‐STAT3, p‐AKT^473^, p‐AKT^308^, and AKT was detected by Western blotting. GAPDH served as the loading control.D, ERepresentative histograms (D) and percentage (E) of CD4^+^ T‐cell proliferation at a ratio of 2:1, 1:1, and 1:2 CD4^+^ T cells to CD11b^+^ myeloid cells collected from CT26 tumor‐bearing mice at 2 weeks post‐treatment with vehicle or 75 mg/kg TP‐16. All data are presented as mean ± SEM derived from three independent experiments (*n* = 3). A two‐tailed unpaired Student’s *t‐*test was performed, ***P < 0.001. Source data are available online for this figure.

**Figure 5:**
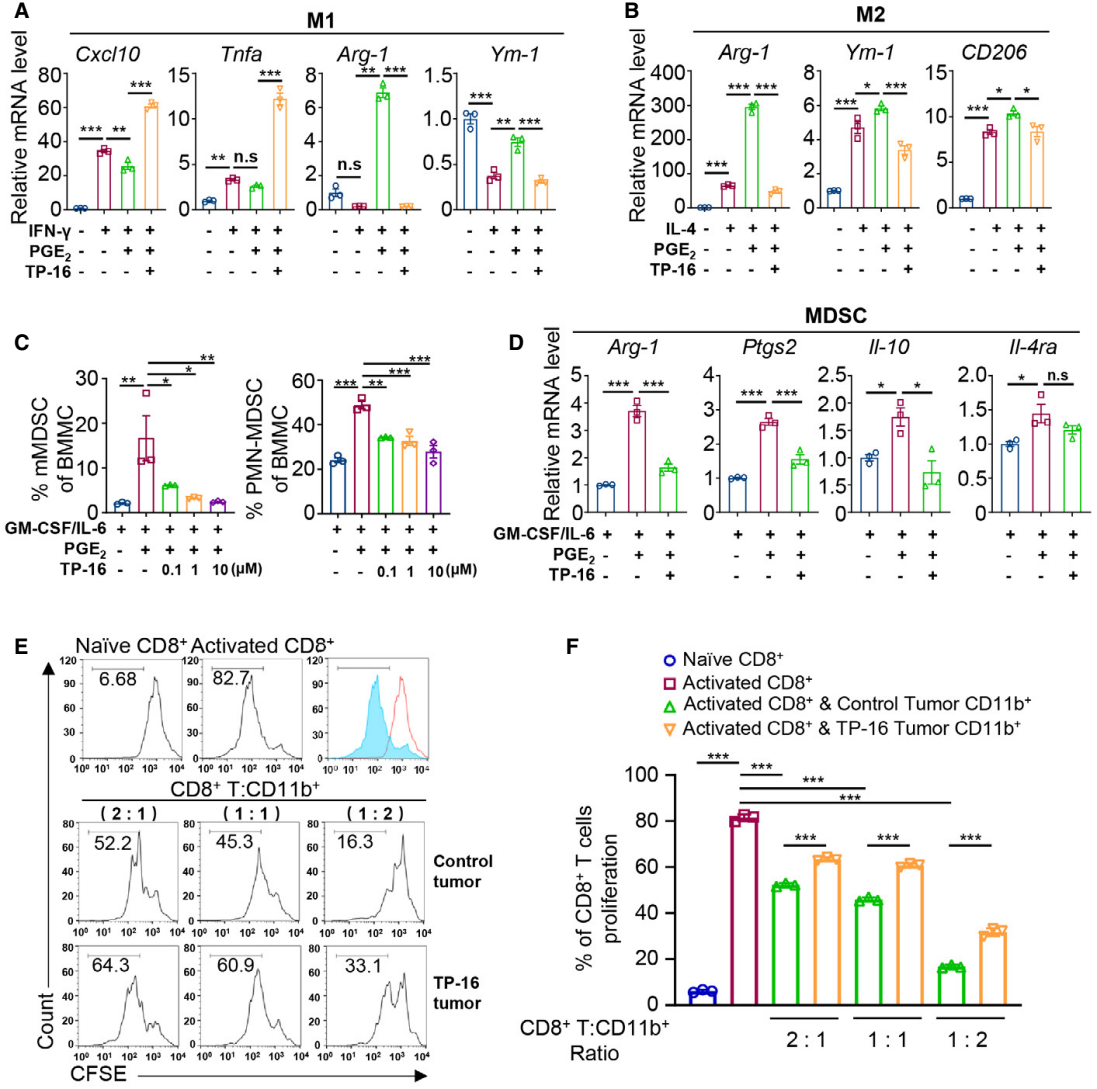
EP4 antagonist TP‐16 reverses the immunosuppressive effects of PGE_2_ on myeloid differentiation and activation A, BM‐CSF‐induced mouse bone marrow‐derived macrophages (BMMs) were stimulated with 50 ng/ml mouse recombinant IFN‐γ (A) or 20 ng/ml mouse recombinant IL‐4 (B) in the absence or presence of PGE_2_ ± varying concentrations of TP‐16 for 12 h. The mRNA levels of selected M1 markers (*Cxcl10* and *Tnfa*) and M2 markers (*Arg‐1* and *Ym‐1*) were measured by qPCR. Data are normalized to β‐actin expression levels (*n* = 3).CBone marrow cells were cultured with 40 ng/ml GM‐CSF/IL‐6 or co‐stimulated with PGE_2_ ± varying concentrations of TP‐16 for 6 days. Representative quantification of Ly6C^+^Ly6G^−^ monocytic myeloid‐derived suppressor cells (mMDSCs) and Ly6C^mid^Ly6G^+^ PMN‐MDSCs (*n* = 3).DBone marrow cells were treated with GM‐CSF/IL‐6 for 6 days to induce MDSCs and thereafter with PGE_2_ ± varying concentrations of TP‐16 for 12 h. The mRNA levels of selected MDSC markers (*Arg‐1, Ptgs2, Il‐4,* and *Il‐10*) were measured by qPCR. Data are normalized to β‐actin expression levels (*n* = 3).E, FIn vitro T‐cell suppression activity of CT26‐ tumor‐infiltrating CD11b^+^ myeloid cells collected at 2 weeks post‐treatment with vehicle or 75 mg/kg TP‐16. CD8^+^ T cells were labeled with CFSE and then stimulated with CD3/CD28 antibodies for 3 days in the absence or presence of tumor‐infiltrating CD11b^+^ myeloid cells from vehicle‐ or 75 mg/kg TP‐16‐treated CT26 tumors. Representative flow cytometry histograms (E) and percentages (F) of proliferating CD8^+^ T cells when plated in a ratio of 2:1, 1:1, and 1:2 CD8^+^ T cells to CD11b^+^ myeloid cells (*n* = 3). Data information: Data are presented as mean ± SEM. (A‐D, F) One‐way analysis of variance (s), Tukey's multiple comparison; *P < 0.05; **P < 0.01; ***P < 0.001. Exact *P* values and statistical tests are listed in Appendix Table S8.

We next investigated whether EP4 inhibition by TP‐16 directly regulated IMCs differentiation and activation. For macrophages analysis (De Henau *et al*, 2016; Tan *et al*, 2018), mouse BM cells were polarized into M1 macrophages or M2 macrophages using IFN‐γ or IL‐4, respectively. TP‐16 significantly suppressed PGE_2_‐induced polarization of M2 macrophages in a concentration‐dependent manner (Fig EV3A). Notably, simultaneous TP‐16 treatment was slightly superior to sequential TP‐16 treatment in M2 macrophage generation triggered by PGE_2_. TP‐16 enhanced the expression of prototypic M1 macrophages markers *Cxcl10* and *Tnfa* on M‐CSF/IFN‐γ stimulation (Fig 5A). However, the expression level of M2 markers (*Arg‐1, Ym1,* and *CD206*) was lower in IL‐4‐polarized M2 macrophages after TP‐16 treatment (Fig 5B). Thereafter, we assessed the effect of TP‐16 on MDSC differentiation and expansion using a GM‐CSF/IL‐6/PGE_2_‐derived MDSC differentiation model (Svoronos *et al*, 2017). TP‐16 significantly inhibited PGE_2_‐dependent expansion of both mMDSCs and PMN‐MDSCs (Fig 5C). Furthermore, TP‐16 reduced the expression of several typical MDSCs markers such as *Arg‐1, Ptgs2, Il‐10,* and *Il‐4ra* (Fig 5D).

The STAT3 and AKT pathways play a critical role in regulating myeloid lineage cells (Gabrilovich *et al.*, 2012; Kaneda *et al*, 2016). We further assessed the activation of STAT3 and AKT signaling using Western blotting. The levels of p‐STAT3 and p‐AKT were significantly increased in myeloid cells incubated with IL‐4/PGE_2_ (Fig EV3B and C). Of note, TP‐16 significantly reversed IL‐4/PGE_2_‐induced phosphorylation of STAT3 and AKT.

Subsequently, we evaluated the direct effect of IMCs derived from the control vehicle‐ or TP‐16‐treated CT26 tumor‐bearing mice on naïve T‐cell proliferation *in vitro*. CD8^+^ T cells were isolated from normal BALB/c mice by magnetic bead separation and were labeled with a fluorescent probe (carboxyfluorescein succinimidyl ester [CFSE]). Labeled CD8^+^ T cells were stimulated with CD3/CD28 antibodies for 3 days in the presence of tumor‐infiltrating CD11b^+^ myeloid cells from the control vehicle or TP‐16 treated CT26 tumor‐bearing mice. Notably, CD11b^+^ myeloid cells from the TP‐16‐treated group suppressed proliferation of CD8^+^ T cells to a lesser extent compared with those from the control vehicle‐treated group (Fig 5E and F). Similar results of TP‐16 treatment were observed in CD4^+^ T cells (Fig EV3D and E). In summary, TP‐16 effectively reversed the immunosuppressive capacity of IMCs.

### TP‐16 improves the efficacy of PD‐1 blockade

**Figure 6:**
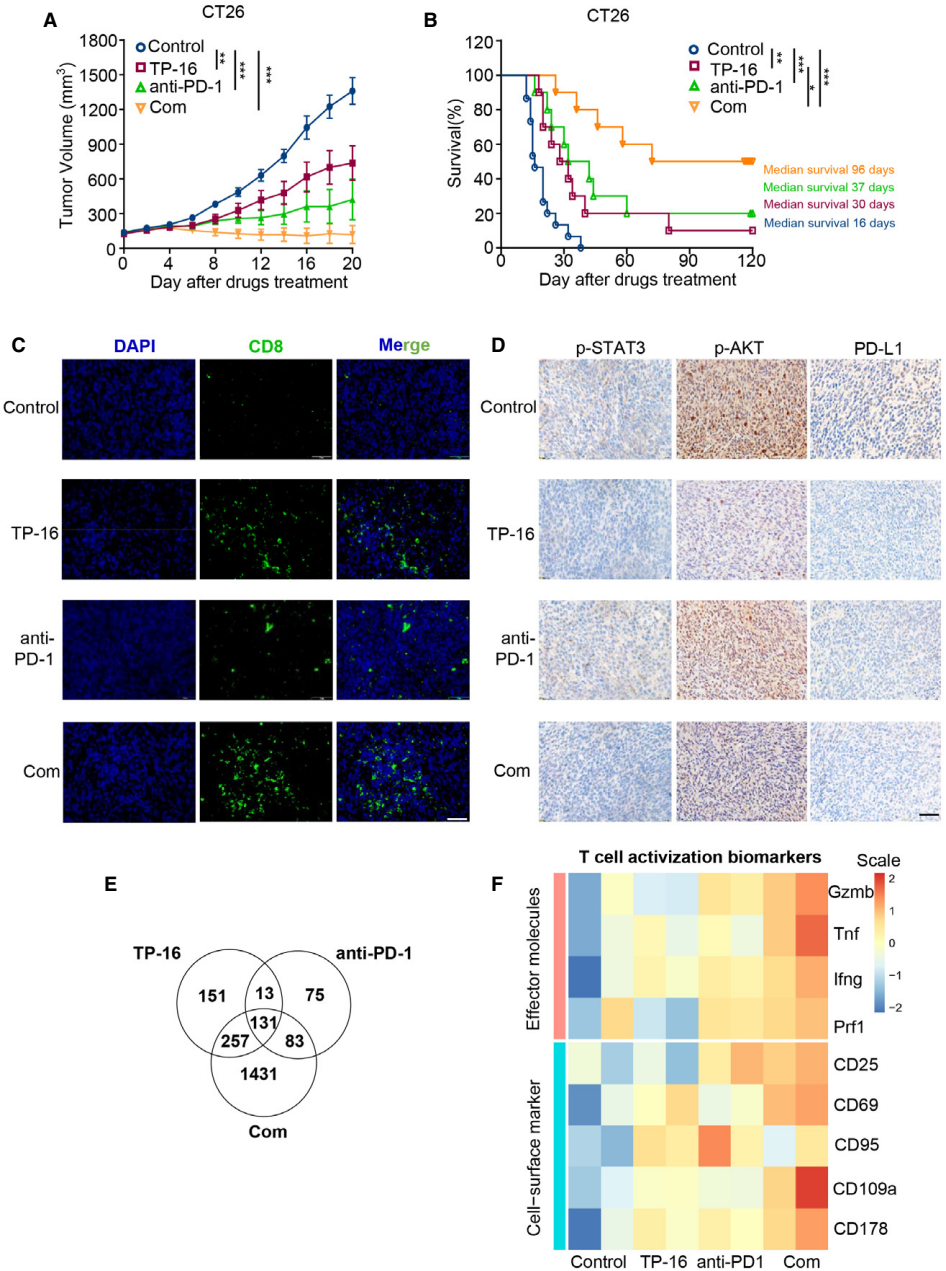
EP4 antagonist TP‐16 improves the efficacy of immune checkpoint blockade and induces a durable anti‐tumor immune response in CT26 tumor‐bearing mice The growth of CT26 tumor volume when treated with vehicle, TP‐16 (75 mg/kg, p.o., daily), anti‐PD‐1 antibody (50 μg, i.p., twice weekly) or their combination (left) and percent change in tumor volumes between days 0 and 20 (right) (*n* = 12).BALB/c mice with subcutaneous CT26 tumors were treated with vehicle, TP‐16 (75 mg/kg, p.o., daily), PD‐1 antibody (50 μg, i.p., twice weekly) or combined therapies for 2 weeks. Animal survival (time to tumor burden reaching 2,000 mm^3^) was analyzed by the Kaplan–Meier method using GraphPad Prism (*n* = 10). *P* values were calculated using the log‐rank test.Representative immunofluorescence staining images of tumor sections from CT26 tumor‐bearing mice stained for CD8 and DAPI. Scale bars, 50 μm.Representative immunostaining images of tumor sections from CT26 tumor‐bearing mice stained for p‐STAT3, p‐AKT, and PD‐L1. Scale bars, 100 μm.Transcriptome profile changes mediated by the synergy of TP‐16 and anti‐PD‐1 analyzed using Agilent SurePrint G3 Mouse GE V2.0 Microarray. Venn diagrams depicting the number of changed genes (FC ≥ 2 or FC ≤ −2, *P* ≤ 0.05) compared with vehicle control following TP‐16, anti‐PD‐1 antibody, or TP‐16 and anti‐PD‐1 combined therapy.The heatmap of T‐cell activation biomarkers for RNA‐seq analysis. The unit of heatmap scale is expression value after centering and scaling by genes. Data information: Data are presented as mean ± SEM. (A) One‐way analysis of variance one‐way analysis (ANOVA), Tukey's multiple comparison; **P < 0.01; ***P < 0.001. (B) log‐rank test; *P < 0.05; **P < 0.01; ***P < 0.001. Exact *P* values and statistical tests are listed in Appendix Table S8. Source data are available online for this figure.

**Figure EV4:**
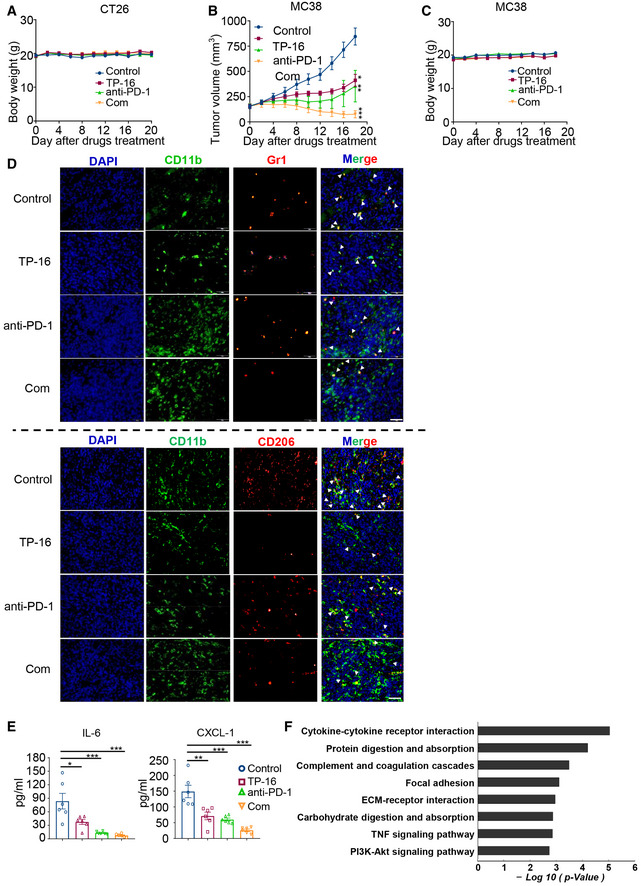
In vivo efficacy of EP4 antagonist (TP‐16) combined with immune checkpoint blockade Body weight of mice (CT26 syngeneic tumor model).The growth of MC38 tumors treated with vehicle, TP‐16 (75 mg/kg, po, daily), anti‐PD‐1 (50 μg, ip, twice weekly), and TP‐16 combined with anti‐PD‐1 antibody (*n* = 8 per group). Data are presented as mean ± SEM. One‐way ANOVA and Tukey's multiple comparison test were performed; *P < 0.05; **P < 0.01; ***P < 0.001.Body weight of mice (MC38 syngeneic tumor model).Representative immunofluorescence staining images of tumor sections from CT26 tumor‐bearing mice stained for myeloid‐derived suppressor cells (MDSCs; CD11b^+^Gr1^+^, upper panel) and M2 macrophages (CD11b^+^CD206^+^, lower panel). Scale bars, 50 μm.Cytokines (IL‐6 and CXCL1) in the peripheral blood of CT26 tumor‐bearing mice treated as indicated were measured by enzyme‐linked immunosorbent assay (ELISA) on the last day of treatment (*n* = 6). One‐way analysis of variance (ANOVA) and Turkey *post hoc* test were performed, *P < 0.05, **P < 0.01; ***P < 0.001.Pathway enrichment was analyzed based on the subsets of differentially expressed genes influenced by the combination therapy of TP‐16 and anti‐PD‐1 antibody. Source data are available online for this figure.

We next asked whether TP‐16 enhanced the response of PD‐1 blockade in syngeneic colon cancer models. Monotherapy with TP‐16 or anti‐PD‐1 inhibited tumor growth at the indicated dosage; however, combination therapy demonstrated a much more potent anti‐tumor efficacy than either monotherapies (Fig 6A). Importantly, the combination of TP‐16 and anti‐PD‐1 remarkably prolonged the survival of tumor‐bearing mice: The median survival times for the control group (*n* = 10), TP‐16 group (*n* = 10), anti‐PD‐1 group (*n* = 10), and combination group were 16, 30, 37, and 96 days, respectively. Of note, 50% of the animals in the combination therapy group (5/10) achieved complete remission. No body weight loss was noted in any of the cohorts, indicating that the dose was well‐tolerated *in vivo* (Fig EV4A). In addition, the combination therapy of TP‐16 and anti‐PD‐1 showed significant anti‐tumor effects in an MC38 colorectal tumor model without systematic toxicity affecting body weight (Fig EV4B and C).

Further, we examined the effects of the combination therapy of TP‐16 and anti‐PD‐1 antibody on the tumor microenvironment. Immunofluorescence analysis of tumor tissues showed that the combination therapy of TP‐16 and anti‐PD‐1 antibody significantly increased the cytotoxic CD8^+^ T‐cell population and reduced MDSCs (CD11b^+^Gr1^+^) and M2 macrophages (CD11b^+^CD206^+^) compared with control vehicle‐ and monotherapy‐treated groups (Figs 6C and EV4D). Tumor‐derived PGE_2_ has been reported to increase the expression of immunosuppressive molecule PD‐L1 in IMCs (Prima *et al*, 2017); however, the involvement of EP subtypes is not defined. Notably, immunohistochemical analysis revealed that TP‐16 monotherapy or the combination therapy of TP‐16 and anti‐PD‐1 antibody effectively repressed PD‐L1 expression and reduced p‐STAT3 and p‐AKT levels, suggesting that EP4 receptor is involved in the regulation of PD‐L1 expression (Fig 6D). Further evidence of reduced immunosuppression following the combination therapy was a significant decrease in pro‐tumor cytokines, such as IL‐6 and CXCL‐1 in the serum of tumor‐bearing mice (Fig EV4E). Overall, TP‐16 facilitated anti‐PD‐1 therapy by reshaping the tumor microenvironment and promoting cytotoxic T‐cell‐mediated tumor regression.

To further explore the mechanisms of these agents, we assessed global transcriptome changes in CT26 tumors isolated from the control vehicle, monotherapy, and combination therapy group mice. We found that the mRNA expression of genes was considerably diverse among the different treatment groups (|FC| ≤ 2, and *P* value ≤ 0.05) (Fig 6E). TP‐16 or anti‐PD‐1 monotherapy changed the gene expression pattern to a certain extent compared with vehicle control treatments, whereas the combination therapy reshaped the gene expression profile of the populations in the tumor microenvironment (Fig 6E). Pathway enrichment analysis revealed that multiple inflammation‐ and immunity‐related pathways, such as cytokine‐cytokine receptor interactions, the TNF signaling pathway, and the PI3K‐AKT signaling pathway, may be responsible for the synergistic effect of TP‐16 and anti‐PD‐1 antibody combination therapy (Fig EV4F). Notably, the combination therapy of TP‐16 and anti‐PD‐1 simultaneously elevated the expression of T‐cell cytolytic effector molecules (*Gzmb*, *Tnfa*, *Ifng,* and *Prf1*) and T‐cell activation cell surface markers (CD25, CD69, CD107a, and CD178) (Fig 6F), suggesting augmented anti‐tumor immunity in a syngeneic colon cancer mouse model.

### TP‐16 renders anti‐PD‐1 antibody effective in AOM/DSS‐induced colorectal cancer model

**Figure 7:**
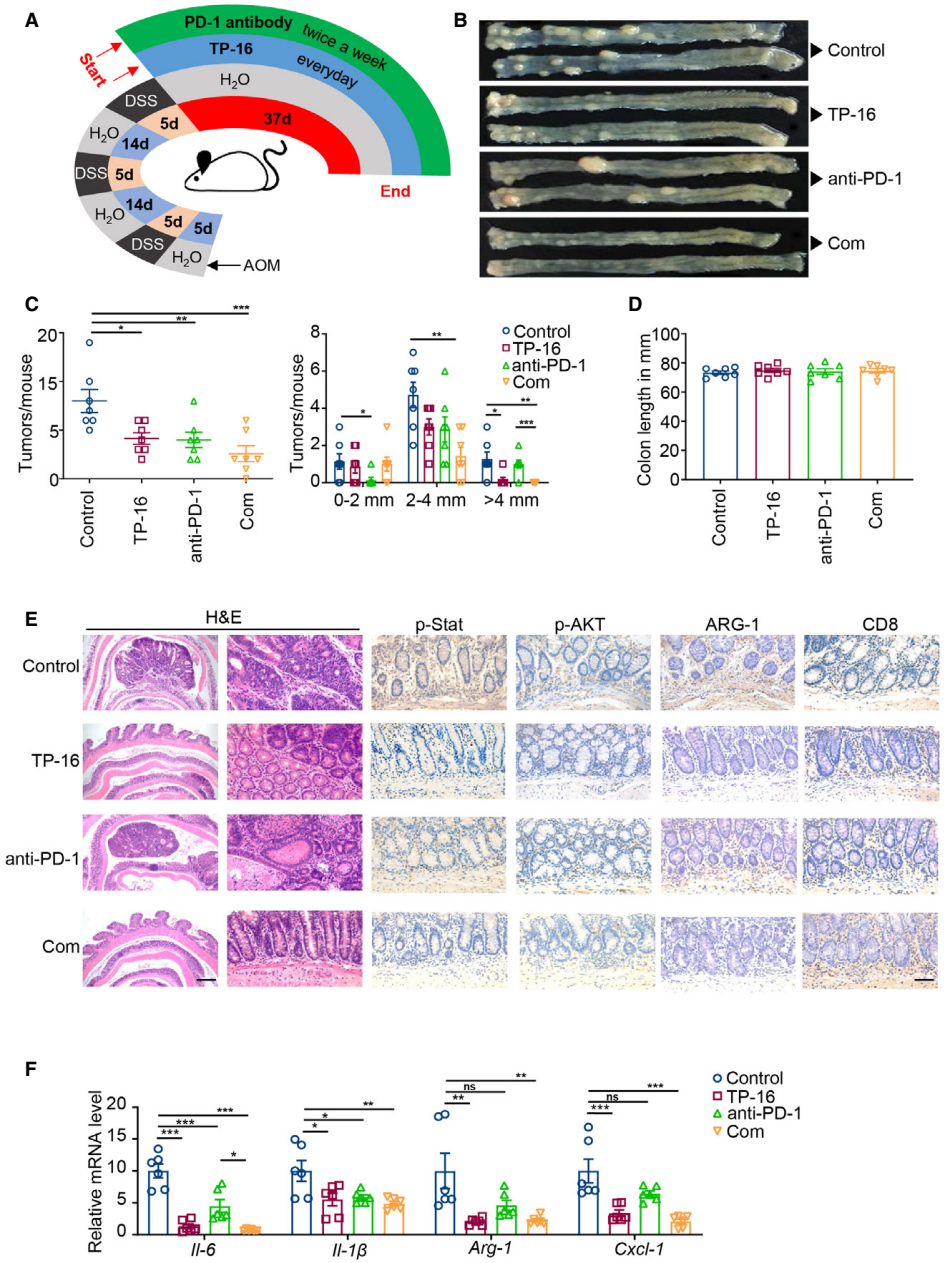
TP‐16 render anti‐PD‐1 antibody effective in AOM/DSS‐induced colorectal cancer model ASchematic diagram of the establishment of azoxymethane/dextran sodium sulfate (AOM/DSS)‐induced colorectal cancer model and drug therapy regimen. AOM/DSS‐induced colorectal tumor mice were treated with vehicle, TP‐16 (75 mg/kg, po, daily), PD‐1 antibody (50 μg, ip, twice weekly), or TP‐16 and anti‐PD‐1 combined. The endpoint was day 37 from treatment initiation.B–DRepresentative resected colons (B), total number of colon tumors per mouse (C, left) and tumor size distribution (C, right), and colon length (D) after 5 weeks of indicated treatment (*n* = 7).ERepresentative hematoxylin and eosin (H&E) staining and immunostaining for CD8, p‐STAT3, p‐AKT, and ARG‐1 of tumors treated with vehicle, TP‐16, anti‐PD‐1 antibody, or TP‐16 and anti‐PD‐1 combined. Scale bars, 500 μm (left images), 50 μm (right images).FQuantitative polymerase chain reaction (qPCR) analysis of the mRNA level of genes associated with immunosuppression (*n* = 6). Data information: Data are presented as mean ± SEM. (C, left panel) One‐way analysis of variance (ANOVA), Tukey's multiple comparison; **P < 0.01; ***P < 0.001. (C, right panel) two‐tailed unpaired Student’s *t*‐test; *P < 0.05; **P < 0.01; ***P < 0.001. (F) Exact *P* values and statistical tests are listed in Appendix Table S8. Source data are available online for this figure.

The azoxymethane/dextran sodium sulfate (AOM/DSS) model, a chronic inflammation‐induced colon cancer model with increased PGE_2_ generation and IMCs infiltration, reflects the pathological process of human colorectal cancer (Neufert *et al*, 2007). We investigated the effect of EP4 blockade and anti‐PD‐1 in the AOM/DSS colorectal cancer model (Fig 7A). We found that TP‐16 monotherapy alone moderately reduced the total tumor number and size, whereas anti‐PD‐1 monotherapy failed to eliminate established tumors with a diameter of > 4 mm (Fig 7B and C). Importantly, the combination therapy of TP‐16 and anti‐PD‐1 significantly reduced both tumor multiplicity and size, compared with control vehicle treatment group. This combination therapy was well tolerated with no change in intestinal length (Fig 7D).

Hematoxylin and eosin (H&E) staining showed considerably reduced AOM/DSS‐induced adenocarcinoma lesions after the combination therapy of TP‐16 and anti‐PD‐1. Immunofluorescence analysis of the colorectal samples of AOM/DSS revealed greater CD8^+^ cytotoxic T‐cell infiltration in the intestine after the combination therapy. Importantly, this combination therapy regimen effectively reduced the levels of p‐STAT3, p‐AKT, and immunosuppression marker (ARG‐1 protein) (Fig 7E) and up‐regulated CD8 expression in adenocarcinoma lesions. We further found that the expression of crucial inflammatory cytokines such as *Il‐6, Il‐1β, Arg‐1,* and *Cxcl1* was remarkably downregulated by TP‐16 monotherapy; however, the combination therapy of TP‐16 and anti‐PD‐1 antibody was not noticeably more effective than TP‐16 monotherapy (Fig 7F). Overall, TP‐16 converted the immunosuppressive microenvironment and repressed the resistance of anti‐PD‐1 in AOM/DSS‐treated mice, a chronic inflammation‐driven colorectal cancer model.

## Discussion

Diverse regulatory mechanisms are exploited by cancer cells to establish a strong immunosuppressive tumor microenvironment that supports tumor growth, fuels tumor immune escape, and weakens immunotherapeutic efficacy (Zou, 2005; Fleming *et al*, 2018). IMCs, a group of heterogeneous phagocytic populations derived from the common myeloid progenitor, are key mediators of such immunosuppressive mechanisms in a wide range of cancer types (Sica *et al*, 2012). Therapies aimed at blocking the immunosuppressive functions of IMCs include traditional chemotherapeutic agents such as gemcitabine, 5‐fluorouracil, doxorubicin, and paclitaxel as well as recently found agents that reduce MDSCs levels at the tumor sites and in peripheral lymphoid organs in certain therapeutic schedules (Wang *et al*, 2017). Given the plasticity and complexity of infiltrative myeloid cells in the tumor microenvironment, better strategies are required to target IMCs and to enhance cancer treatment efficacy, particularly tumor immunotherapy (Hossain *et al*, 2015). In this study, we focused on the therapeutic disruption of IMC‐mediated immunosuppression by targeting the PGE_2_‐EP4 signaling pathway in colorectal cancer. Thus, we developed a novel and selective human EP4 antagonist, TP‐16 to block the immunosuppressive effects of IMCs in the tumor microenvironment. Importantly, TP‐16 impaired tumor growth in multiple cancer models, reprogrammed myeloid compartments, and enhanced cytotoxic T‐cell infiltration and activation in tumor tissues.

PGE_2_ is a critical factor in colorectal cancer immunopathology. COX2, which catalyzes the biosynthesis of prostanoids, is overexpressed in most colorectal cancer tissues (Dannenberg & Subbaramaiah, 2003). Emerging evidence has indicated that COX2 overexpression in cancer cells dictates the immune landscape of tumors (Wellenstein & de Visser, 2018). For instance, tumor‐derived PGE_2_ drives cancer progression by impairing natural killer cells and the conventional dendritic cell resulting in cancer immune evasion (Zelenay *et al*, 2015; Bottcher *et al*, 2018). Several oncogenic signaling pathways have been reported to stimulate COX2 transcription (Dannenberg & Subbaramaiah, 2003). In this study, we systematically investigated the effects of a panel of oncogenic mutations on COX2 expression and found that gain‐of‐function of the RAS‐MAPK, PI3K‐AKT, and IL‐6‐JAK‐STAT3 pathway significantly induced COX2 expression (Appendix Fig S3). These data suggest that, at least in certain cancers, PGE_2_ produced by cancer cells may impair anti‐tumor immune responses, highlighting the existence of common immune regulatory mechanisms in tumor progression.

Several studies have shown that COX inhibitors, which include nonsteroidal anti‐inflammatory drugs (NSAIDs), reduce the risk of cancer incidence and death due to advanced malignant diseases such as colorectal cancer (Thun *et al*, 2002; Gurpinar *et al*, 2014). Simultaneously, clinical, and epidemiological data have indicated that prolonged COX‐PGE_2_ inhibition with NSAIDs at high dosages is associated with an increased risk of gastrointestinal and cardiovascular events compared with non‐NSAIDs (Mukherjee *et al*, 2001; Wang & Dubois, 2006; Sostres *et al*, 2010). Recently, Deng et al. reported that a prostaglandin E Synthase inhibitor (Cay10526) suppressed tumor malignant progression in a spontaneous lung adenocarcinoma model (Wang *et al*, 2020); however, its clinical benefits require further investigation. To date, several EP4 antagonists have entered human clinical trials for the treatment of migraine, inflammatory diseases, and cancer. Grapiprant is the most advanced EP4 antagonist (phase II) for the treatment of advanced solid tumors; however, its unsatisfactory pharmacokinetic profiles may hinder its clinical efficacy (Markovic *et al.*, 2017). In this study, TP‐16 was identified as a novel, potent, and selective EP4 antagonist with an excellent safety profile and pharmacokinetic properties that promoted T‐cell‐dependent tumor elimination by reprogramming IMCs. Furthermore, even at a lower dosage, TP‐16 was superior to celecoxib, a COX‐2 inhibitor. Overall, these data suggest that EP4 antagonists can be a good alternative to NSAIDs for cancer therapy as immunological adjuvants.

EP2 and EP4, two GPCRs, are considered executors of immune modulation activity of PGE_2_ (Kalinski, 2012). In this study, EP4, rather than EP2, was identified as the major receptor regulating the differentiation and expansion of IMCs. We reason that the discrepancy at certain stages may be caused by the differential cellular and tissue expression of EP2 and EP4. For instance, several previous studies have shown that EP4 is the prominent prostaglandin receptor in human and mouse macrophages (Nataraj *et al*, 2001; Takayama *et al*, 2002). Furthermore, a specific role of EP4, but not of other EP subtypes, has been reported in human and mouse‐derived myeloid cell differentiation and function (Chang *et al*, 2015; Albu *et al*, 2017). However, TAMs and MDSCs are the main target cells of TP‐16, and the PGE_2_‐EP4 signaling pathway facilitates cancer‐promoting immunoreaction in other immune cells, such as suppression of natural killer cell activity and induction of Treg cell development (O'Callaghan & Houston, 2015). Future investigations will clarify whether these immune cells contributed to the anti‐tumor efficacy TP‐16.

Colorectal cancer, especially the MMS genotype, is largely resistant to immunotherapy (Le *et al.*, 2017). IMCs, including TAMs and MDSCs, are the major drivers of an immunosuppressive tumor microenvironment and can contribute to resistance to ICB therapy (Highfill *et al*, 2014; De Henau *et al.*, 2016), highlighting the potential for further improvements by combining specific IMC‐targeted therapeutics. Several groups have reported that the inhibition of myeloid PI3K can switch the suppressive status of IMCs toward an inflammatory phenotype and overcome ICB resistance (De Henau *et al.*, 2016; Kaneda *et al.*, 2016). This combination strategy has been evaluated in several clinical trials. Recently, oncogenic KRAS was found to promote an immunosuppressive tumor microenvironment through the CXCL3‐CXCR2 pathway in MDSCs, providing a potential combination therapeutic strategy for the treatment of advanced colorectal cancer (Liao *et al.*, 2019). Our data demonstrate that the EP4 antagonist (TP‐16) synergistically acts with PD‐1 antibody to target IMCs in CT26 and MC38 syngeneic mouse models. However, these two cell lines have high tumor mutation burden (TMB), which may not reflect the pathological features and clinical responses of colorectal cancer patients with low TMB (mainly MSS‐type). Notably, two clinical trials of EP4 antagonists (Grapiprant and AN0025) combined with immune checkpoint therapy (pembrolizumab) in patients with advanced or progressive MSS colorectal cancer are currently under way (Grapiprant: NCT03658772; AN0025: NCT04432857). These studies will soon reveal the therapeutic potential of EP4 antagonist and anti‐PD‐1 combination for MSS colorectal cancer in clinical settings.

Our mechanistic study demonstrated that TP‐16 and anti‐PD‐1 synergistically reduced the serum level of IL‐6, a critical immunosuppressive factor, in tumor‐bearing mice. Moreover, TP‐16 monotherapy or the combination therapy of TP‐16 and anti‐PD‐1 effectively suppressed PD‐L1 expression in tumor tissues. In line with these observations, increased infiltration and activation of CD8^+^ T cells in tumor tissue was observed in the combination therapy group. Although the detailed molecular mechanism of the synergistic effect is unclear, our findings offer a rationale for exploring EP4 inhibition to sensitize patients to ICB therapy in clinical trials.

In summary, EP4 inhibition by TP‐16 can boost T‐cell‐mediated anti‐tumor immunity by targeting the IMC‐mediated immunosuppressive tumor microenvironment. Oral administration of TP‐16 facilitates the anti‐tumor treatment of ICB therapy with good tolerance and safety. The comprehensive preclinical data presented here suggest that TP‐16 provides a framework for the combination therapy of EP4 antagonists and T‐cell‐targeting agents in colorectal oncology.

## Materials and Methods

### Cell culture

The murine colon cancer (CT26), murine breast cancer (4T1), and human colon cancer (HCT116, HCT8, HT29, and DLD1) cell lines were obtained from American Type Culture Collection (ATCC). The murine cell lines of melanoma (B16F10), pancreatic cancer (Pan02), and colon cancer (MC38), Chinese hamster ovary (CHO) cell line, and human embryonic kidney cell lines (HEK293 and HEK293T) were purchased from the National Infrastructure of Cell Line Resource (Shanghai, China). Primary human endothelial cells (HUVECs) were kindly provided by Dr. Xinli Wang (Baylor College of Medicine, Houston, TX, USA) and cultured in endothelial cell medium (ScienCell, San Diego, CA, USA) according to the manufacturer’s instructions. The CHO‐Gα16 cell line was established in our previous study and maintained in DMEM/F12 medium containing puromycin (4 μg/ml) (Wu *et al.*, 2018). Cell lines were maintained in vendor recommended growth medium supplemented with 10% FBS (Gibco, Waltham, MA, USA) and 1% penicillin/streptomycin solution (Gibco) at 37°C ins a humidified atmosphere (5% CO_2_). All cell lines were authenticated using short tandem repeat analysis. All cell lines were confirmed to be mycoplasma‐free by polymerase chain reaction (PCR) analysis. Cell viability was determined using CellTiter 96 ® Aqueous One Solution (Promega, Madison, WI).

### Animal models

BALB/c, BALB/c nude, C57BL/6, and CD‐1 female mice (6‐10 weeks old) were purchased from the National Rodent Laboratory Animal Resources (Shanghai, China). All mice were group‐housed under specific pathogen‐free conditions at an appropriate temperature (20‐22°C) and humidity (60%), and a 12 h light/dark cycle. Water and standard pelleted food were provided *ad libitum*. All animal studies were performed using a protocol approved by the East China Normal University Ethics Committee.

Syngeneic mouse tumor‐bearing models were established as described previously (Tan *et al.*, 2018). Tumor cells (5 × 10^5^ ‐ 2 × 10^6^) were suspended in 100 μl PBS and implanted subcutaneously into the flank of mice. Mice were randomized into different treatment groups when tumor volumes reached 100‐200 mm^3^. V = L×W^2^ × 0.52, where L is the longest diameter, and W is the shortest diameter. Tumor size and body weight were recorded every second or third day. Mouse survival was analyzed using the Kaplan–Meier method.

An orthotopic colon cancer model was established as described previously (Hite *et al*, 2018; Song *et al*, 2018). In brief, 6‐week‐old female BALB/c mice were anesthetized using 2.5% isoflurane and properly positioned. A 2‐3 cm incision was made in the skin of the abdominal cavity, and the cecal wall and cecum were injected with CT26‐Luc cells (1 × 10^6^). The wound of the abdominal wall was closed by suture. The tumor growth in each mouse was monitored 5 min after intraperitoneal injection of D‐luciferin (3 mg/mouse) using an IVIS Spectrum imaging system.

For the AOM/DSS‐induced murine colorectal carcinoma model, 10‐week‐old female C57BL/6 mice were given a single injection of AOM (10 mg/kg in PBS; i.p.; Sigma‐Aldrich, St. Louis, MO, USA). After 1 week of rest, mice were provided with 2.5% DSS (MP Biomedicals, Santa Ana, CA, USA) in their drinking water for 5 days, followed by DSS‐free water for two weeks. The DSS treatment cycle was carried out three times in total, and mice were randomized into four groups and subjected to the therapeutic protocol. Mice were sacrificed at 37 days after the indicated treatments.

Single agent or combination treatment was designed as follows: TP‐16 (37.5, 75, 150 mg/kg) or celecoxib (100 mg/kg) was formulated in 0.5% carboxymethylcellulose sodium (CMC‐Na, Sangon Biotech, Shanghai, China) and administered via oral gavage daily. *In vivo* anti‐PD1 antibody (clone RMP1‐14, BioXCell, West Lebanon, NH, USA) and rat IgG2a isotype control (clone 2A3, BioXCell) were diluted in sterile PBS and administered intraperitoneally at a dose of 50 μg twice weekly.

### FACS analysis

Tumor tissues were collected, minced into small pieces, and digested with collagenase I (10 U/ml, Gibco, Waltham, MA), collagenase IV (400 U/ml, Gibco) and DNase I (100 μg/ml, Roche, Basel, Switzerland) in FBS‐free RPMI 1640 medium for 30 min at 37°C. Digested tumor tissues were filtered through 40‐μm cell strainers (Falcon, San Jose, CA, USA) to generate single‐cell suspensions. Spleen single‐cell suspensions were prepared by grinding the spleen through 40‐μm cell strainers (Falcon). After red blood cell lysis, the suspensions were washed with PBS containing 1% FBS and 2 mM EDTA prior to staining with anti‐CD16/32 FcR blocking antibody (Clone 93, BioLegend). Cell surface staining was performed for 30 min at 4°C. For intracellular staining of IFN‐γ, GzmB, and TNF‐α in CD8^+^ T cells, cells were further stimulated with leukocyte activation cocktail (BD Biosciences, San Jose, CA, USA) for 6 h. For intracellular staining of TNF‐α in CD11b^+^ myeloid cells, 100 ng/ml lipopolysaccharide (LPS, Sigma‐Aldrich) was added for 6 h before cell surface staining. Fixation and permeabilization were performed using the fixation/permeabilization kit (BD Biosciences) for 20 min according to the manufacturer’s instructions before staining with intracellular marker antibodies for 45 min at 4°C. All data acquisition was performed using LSR Fortessa or FACSCalibur cell analyzer (BD Biosciences), and analyzed by FlowJo software (TreeStar Inc., Ashland, OR).

The following fluorophore‐conjugated antibodies (1:100 dilution) were used for FACS analysis: anti‐mouse CD45‐PerCP/cy5.5 (Clone C30‐F11, BioLegend), CD8‐APC (Clone 53‐6.7, BioLegend), IFN‐γ‐PE (Clone XMG1.2, BD Biosciences), GzmB‐PE (Clone NGZB, eBioscience, San Diego, CA, USA), TNF‐α‐PE (Clone MP6‐XT22, BioLegend), CD4‐APC (Clone GK1.5, BioLegend), CD4‐ PerCP/cy5.5 (Clone GK1.5, BioLegend), CD11b‐FITC (Clone M1/70, BioLegend), F4/80‐APC (Clone BM8, BioLegend), CD11c‐PE (Clone N418, BioLegend), CD11c‐APC (Clone N418, BioLegend), Ly6C‐PE (Clone HK1.4, BioLegend), Ly6G‐PerCP/cy5.5 (Clone 1A8, BioLegend), Ly6G‐APC (Clone 1A8, BioLegend), MHC‐II‐PE (Clone M5/114.15.2, BioLegend), CD206‐PE (Clone C068C2, BioLegend), and PD‐1‐PE (Clone 29F.1A12, BioLegend).

### Immunohistochemistry and immunofluorescence analysis

Formalin‐fixed paraffin‐embedded tissues from the CT26 tumor model and AOM‐DSS mice were sectioned at 10 μm thickness. After blocking, the sections were incubated with specific primary antibodies followed by incubation with secondary antibodies conjugated with horseradish peroxidase or fluorescent dye. All immunohistochemistry images were acquired using the OLYMPUS CellSens Standard 1.18 system. The primary antibodies used in immunohistochemistry were as follows: CD8 (1: 200 dilution, ab33786, Abcam, Cambridge, UK), Granzyme B (1:100 dilution, ab4059, Abcam), PD‐1 (1:100 dilution, GB13338, Servicebio, Hubei, China), CD11b (1:100 dilution, Ab133357, Abcam), Gr1 (1:50 dilution, Mab1037, R&D system, Minneapolis, MN, USA), CD206(1:100 dilution, ab64693, Abcam), p‐CREB (1:200 dilution, S113, ab32096, Abcam), p‐Stat3 (1:100 dilution, Tyr705, 9145, CST, Danvers, MA), p‐Akt (1:100 dilution, Ser473, 4060, CST), and Arg‐1 (1:200 dilution, 16001, Proteintech, Rosemont, IL, USA).

### *In vitro* monocyte‐derived functional immune assay

Single‐cell suspensions of mouse BM cells were prepared by filtering femurs and tibias of 6‐week‐old female BALB/c mice through 40‐μm cell strainers (Falcon). These suspensions were treated with red blood cell lysis buffer and replated in conditioned medium. Monocyte differentiation, MDSC induction, and macrophage polarization were analyzed by FACS analysis (BD Biosciences) with FlowJo software.

For the mouse monocyte differentiation assay (Helft *et al*, 2015), purified BM cells were differentiated in RPMI1640 medium containing recombinant mouse GM‐CSF (20 ng/ml, Peprotech, Rocky Hill, NJ, USA) and IL‐4 (10 ng/ml, Peprotech) in the absence or presence of PGE_2_ (10 nM, Cayman Chemical) for 9 days at 37°C. The medium was refreshed every 3 days. Meanwhile, EP1 antagonist (ONO‐8711, 10 μM, Cayman Chemical), EP2 antagonist (PF‐04418948, 10 μM, TopScience, Shanghai, China), EP3 antagonist (L‐798106, 10 μM, Sigma‐Aldrich), and EP4 antagonist (E7046, 10 μM) were added simultaneously.

For the MDSC induction assay, purified BM cells were cultured in RPMI1640 medium stimulated with recombinant mouse GM‐CSF (40 ng/ml, Peprotech) and IL‐6 (40 ng/ml, Peprotech) for 6 days to generate bone marrow‐derived MDSCs (Svoronos *et al.*, 2017); the medium was refreshed every 3 days. Vehicle or PGE_2_ (10 nM) ± TP‐16 was added simultaneously on day 0 for FACS analysis of BM‐derived MDSCs. For quantitative PCR (qPCR) analysis of MDSCs, differentiated MDSCs were treated with vehicle or PGE_2_ (10 nM) ± TP‐16 for 12 h.

For macrophage polarization assay (De Henau *et al.*, 2016; Tan *et al.*, 2018), mouse BM cells were cultured in RPMI1640 medium supplemented with recombinant mouse M‐CSF (20 ng/ml, Peprotech) to generate BM‐derived macrophages (BMDM). For FACS analysis of BM‐derived M2‐like macrophage, IL‐4 (20 ng/ml, Peprotech) and PGE_2_ (10 nM) were added on day 0. TP‐16 was added simultaneously with PGE_2_ on day 0 or sequentially on day 3 for differentiation over a period of 6 days. For qPCR analysis of macrophages (day 6), BMDMs were polarized with IFNγ (50 ng/ml, Peprotech) or IL‐4 (20 ng/ml, Peprotech) ± PGE_2_ (10 nM) ± TP‐16 for 24 h at 37°C on day 6. The culture medium was refreshed every 3 days.

### Isolation of immune cell subsets from tumor tissues

Tumor single‐cell suspensions were prepared as described in the aforementioned section. CD11b^+^ myeloid cells were isolated from tumors using a CD11b^+^ cell separation kit (Miltenyi Biotec, Germany). For FACS sorting of immune cells from tumors, tumor single‐cell suspensions were stained with the following cell surface markers as indicated: anti‐CD11b‐FITC, F4/80‐APC, Ly6C‐PE, Ly6G‐PerCP/cy5.5, MHC‐II‐PE, and CD206‐PE antibodies. FACS sorting was performed on a FACS Aria II Cell Sorter (BD Biosciences). The purity of the flow‐sorted population was above 90%.

### *In vitro* T‐lymphocyte proliferation assay

T cells were isolated from the spleens of naïve BALB/c mice (T‐cell isolation kit, BioLegend). T cells (1 × 10^5^) were labeled with 3 μM carboxyfluorescein succinimidyl ester (CFSE; Thermo Fisher Scientific, Waltham, Massachusetts, USA) and co‐stimulated with anti‐CD3 (2 μM, clone 17A2, BioLegend) and anti‐CD28 (6 μM, clone 37.51, BioLegend) in 96‐well plates. Thereafter, T cells were co‐cultured with CD11b^+^ myeloid cells isolated from tumors (CD11b^+^ cell separation kit, Miltenyi Biotec) at different ratios. After 72 h, T‐cell proliferation was quantified using CFSE dilution by FACS analysis (BD Biosciences) and analyzed by FlowJo software.

### qPCR

Total mRNA was isolated from immune and tumor cells using the TRlzol method (TaKaRa, Shimogyo‐ku, Kyoto, Japan), and cDNA was synthesized from 1 μg RNA using the PrimeScript cDNA synthesis kit (TaKaRa). The EZ‐press Cell to cDNA Kit PLUS kit (EZBioscience, Roseville, MN) was used to isolate total mRNA from sorted immune cell subsets. A SYBR Green‐based qPCR (Yeasen, Shanghai, China) assay was performed using murine primers for *Ptger1, Ptger2, Ptger3, Ptger4, Cxcl10, Tnfa, Ccl2, Ccl5, Arg‐1, CD206, Fizzl, Ym1, Ptgs2, Il‐4rα, Ido1, Il‐10, Il‐6, Il‐1β*, and *Il‐10*. The mRNA levels of target genes were normalized to β‐actin expression using the _ΔΔ_Ct method. The primers sequences are listed in Appendix Table S7.

### Western blot analysis

Briefly, cells and tumor tissues were lysed with radio immunoprecipitation assay buffer containing phosphatase and protease inhibitor cocktail (MedChemExpress, Monmouth Junction, NJ, USA). A total of 30‐80 μg of normalized total proteins from lysates were run on 6‐10% SDS‐polyacrylamide gels. The proteins transferred from gels to nitrocellulose membranes (Millipore, Billerica, MA, USA) were incubated with primary antibodies overnight at 4°C, washed, and incubated with secondary antibodies for 1 h at room temperature. Visual signals were detected using the LI‐COR Odyssey Infrared Imaging System (LI‐COR Biosciences, Lincoln, NE). Mouse protein primary antibodies used for Western blotting were listed as follows: COX2 (1:1000 dilution, 12282, CST), p‐Stat3 (1:2000 dilution, Tyr^705^, 9145, CST), Stat3 (1:1000 dilution, 9139, CST), p‐Akt (1:2000 dilution, Ser^473^, 4060, CST), Akt (1:1000 dilution, 9272, CST), Arg‐1 (1:100 dilution, 16001, Proteintech, Rosemont, IL), and GAPDH (1:10000 dilution, Ab181602, Abcam).

### ELISA assay

For mouse peripheral blood cytokine and chemokine measurements, CT26 tumor‐bearing mice were treated with TP‐16 or anti‐PD‐1 antibody, or both for 2 weeks. Peripheral blood was collected on day 14, and plasma was centrifuged at 8000 rpm for 15 min. Subsequently, supernatants were analyzed using ELISA kits for IL‐6 (BioLegend, San Diego, CA) and CXCL1 (Abnova, Walnut, CA) according to the manufacturer’s instructions.

### Microarray analysis

CT26 tumor‐bearing mice were treated with vehicle, TP‐16, anti‐PD‐1 antibody (75 mg/kg, p.o., daily), or TP‐16 plus anti‐PD‐1 antibody (50 μg, i.p., twice weekly) for 2 weeks. Total RNA from tumor tissues was extracted using the TRlzol method (TaKaRa). Total RNA was quantified using a NanoDrop ND‐2000 (Thermo Fisher Scientific) and assessed using an Agilent Bioanalyzer 2100 (Agilent Technologies, Santa Clara, CA). Microarray analysis was performed using the Agilent SurePrint G3 Mouse GE V2.0 (Agilent Technologies) according to the manufacturer’s protocol.

Raw data were analyzed and normalized using Genespring (version 13.1, Agilent Technologies). Different treatment groups were compared by relative fold change (|FC| ≥ 2), and *P* value (*P* ≤ 0.05) was calculated using the Student’s *t*‐test. Furthermore, gene ontology (GO) enrichment, Kyoto Encyclopedia of Genes and Genomes (KEGG) enrichment, and hierarchical clustering analysis of differentially expressed genes were performed. The techniques and methods for microarray analysis were provided by OE Biotech (Shanghai, China).

### CRE reporter assay

HEK293 cells overexpressing a CRE luciferase reporter were plated in 48‐well plates at a density of 2.5 × 10^4^ cells/200 μl per well and maintained overnight. On the following day, cells were starved for 2 h and then stimulated with 10 nM PGE_2_ in the absence or presence of various concentrations of TP‐16 for 24 h. Luminescence was determined using a dual‐luciferase assay kit (Promega) with a Cytation 5 imaging reader (BioTek).

### Calcium flux analysis

Briefly, CHO‐Gα16 cells individually overexpressing EP1 (human), EP2 (human), EP3 (human), and EP4 (human, monkey, dog, rat, and mouse) were plated into 96‐well‐black plates at a density of 2 × 10^4^ cells/100 μl per well and incubated overnight. On the following day, the plate was loaded with the reagents (100 μl/well) of the Calcium‐5 Assay Kit (Molecular Devices, San Jose, CA, USA) for 45 min at 37°C according to the manufacturer’s instructions. After pretreating with varying concentrations of TP‐16 for 15 min at room temperature, cell plates were treated with PGE_2_ (EC_80_) and analyzed using the Flexstation® 3 Multi‐Mode Microplate Reader (Molecular Devices). Florescence measurement data were continuously recorded for 90 s (Ex wavelength = 485 nm; Em wavelength = 525 nm).

### cAMP glosensor assay

HEK293 cells were co‐transfected with the pGloSensor™‐22F cAMP plasmid and human EP4 overexpressing plasmid (1:1) and maintained overnight. On the following day, cells were replated into 384‐well white plates at a density of 2 × 10^4^ cells/20 μl per well in equilibration with CO_2_‐independent medium (Gibco) containing a 4% v/v dilution of GloSensor™ cAMP Reagent stock solution (Promega). After incubation for 1.5 h at room temperature, cells were pretreated with varying concentrations of TP‐16 for 30 min and then simulated with PGE_2_ (0.1 nM). Luminescence kinetic measurements for 30 min were acquired using a Cytation 5 imaging reader (BioTek, Winooski, VT).

### β‐arrestin Tango assay

CHO cells were co‐transfected with β‐arrestin‐tobacco etch virus (TEV), tTA‐Luc, and EP4‐Tango plasmids (1:2:2), plated into 96‐well plates at a density of 2 × 10^4^ cells/100 μl per well, and maintained overnight. On the following day, cells were starved for 5 h and then stimulated with 10 nM PGE_2_ in the absence or presence of various concentrations of TP‐16 for 16 h. Luminescence was determined using a dual‐luciferase assay kit (Promega) with a Cytation 5 imaging reader (BioTek).

### Data and statistical analysis

Data are presented as mean ± standard error of mean (SEM) and analyzed using GraphPad Prism Software version 8.0 (GraphPad). The information of the experimental groups was blinded to the researchers for histological analysis and animal studies. To compare two treatment groups, statistical significance was determined by a two‐tailed unpaired Student’s *t*‐test; to compare multiple treatment groups, one‐way analysis of variance (ANOVA) followed by Tukey’s multiple comparisons test was performed. A *P* value of < 0.05 was considered statistically significant (*P < 0.05; **P < 0.01; ***P < 0.001).

### Reagents

The detailed information of reagents is provided in Appendix Table S9.

## Author contributions

WQL and MYL conceived the project. WWY, WQL, JCH, WJL, and XHL conducted the main cell biology experiments and mouse experiments. JJY and HKZ conducted the synthesis of EP4 antagonist TP‐16. QSZ and HYY conducted the molecular simulation. WHJ and JL performed the 4T1 mouse experiments. YJZ and XW conducted the pharmacokinetics study. SHP, ZFY, SCR, and JC provided some experiments materials. WQL, WWY, and MYL wrote the manuscript with input from FXC, SS, and RN All authors reviewed and edited the manuscript.

## Conflict of interest

The authors declare that they have no conflict of interest.

## For more information

Clinical trials using EP4 antagonists for cancer therapy.

- Grapiprant (NCT03658772): https://clinicaltrials.gov/ct2/show/NCT03658772
- AN0025 (NCT04432857):https://clinicaltrials.gov/ct2/show/NCT04432857

## Supporting information



Appendix

Expanded View Figures PDF

Source Data for Expanded View and Appendix

Review Process File

Source Data for Figure 3

Source Data for Figure 6

Source Data for Figure 7

## Data Availability

The RNA‐seq datasets generated in this work are available at National Centre for Biotechnology Information Gene Expression Omnibus website under the accession number GSE132004, (https://www.ncbi.nlm.nih.gov/geo/query/acc.cgi?acc=GSE132004).
