# Supplementary material for: Reprogramming immunosuppressive myeloid cells facilitates immunotherapy for colorectal cancer
Source: EMBO Mol Med. 2020 Dec 7;13(1):e12798. doi: 10.15252/emmm.202012798 (PMC7799360; doi:10.15252/emmm.202012798)
Supplement: Supplementary file 1 — Appendix [file EMMM-13-e12798-s001.pdf]

Reprogramming Immunosuppressive Myeloid Cells Facilitates Immunotherapy for Colorectal Cancer

Weiqiang Lu[1,#,*], Weiwei Yu[1,#], Jiacheng He[1], Wenjuan Liu[1], Junjie Yang[1], Xianhua Lin[1], Yuanjin Zhang[1], Xin Wang[1], Wenhao Jiang[1], Jian Luo[1], Qiansen Zhang[1], Huaiyu Yang[1], Shihong Peng[1], Zhengfang Yi[1], Shancheng Ren[2], Jin Chen[3], Stefan Siwko[4], Ruth Nussinov[5,6], Feixiong Cheng[7-9], Hankun Zhang[1,*], Mingyao Liu[1,*]

Appendix

Appendix Materials and Methods (Pages 2-5)
Appendix Figures S1-S3 (Page 6-8)
Appendix Tables S1-S9 (Page 9-29)

Appendix Materials and Methods

Chemistry: Preparation of TP-16

Reagents and solvents were obtained from commercial sources and used without further purification. All reactions were carried out with the use of standard techniques under an argon atmosphere and the progress of the reactions was monitored by TLC on SiO_2. Proton and carbon NMR spectra were recorded at spectrometer frequencies of 500 MHz and obtained as $CDCl_3$ or DMSO-d_6 solutions (reported in ppm), using $CDCl_3$ (7.26 and 77.00 ppm) or DMSO-d_6 (2.50 and 39.51 ppm) as the reference standard. High-resolution mass spectra (HRMS) were gathered on a Bruker MicroTOF-Q II LCMS instrument operating in electrospray ionization (ESI). HPLC (Agilent Technologies 1200 Series) was employed for purity determination, using the following method: SunFire C18 column, 5 µm, 4.6x150 mm; column temperature 40 °C; with detection at 254 or 214 nm on a variable wavelength detector; flow rate = 1.0 mL/min; gradient of 0−100% acetonitrile in water (both containing 0.03 vol% of CF_3COOH) in 16 min.

Synthesis of TP-16[a]

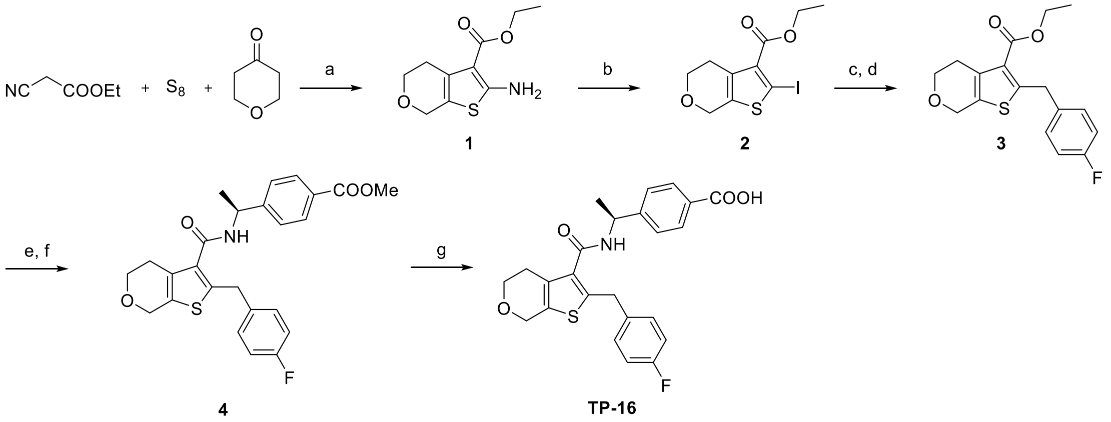

[a]Reagents and conditions: (a) morpholine, EtOH, 60 °C, 10 h, 90.5%; (b) 3M HCl (aq), $NaNO_2$, KI, H_2O, 0 °C, 34.9%; (c) n-BuLi (2.4 M in hexane), 4-fluorobenzaldehyde, Et_2O, -78 °C to room temperature (rt), 50.7%; (d) Et_3SiH, TFA, CH_2Cl_2, 0 °C, 95%; (e) LiOH·H_2O, MeOH, THF, H_2O, 70 °C, 100%; (f) methyl 4-[(1S)-1-aminoethyl]benzoate, 2-(7-azabenzotriazol-1-yl)-N,N,N',N'-tetramethyluronium hexafluorophosphate (HATU), DIPEA, DMF, rt, 82%; (g) LiOH·H_2O, MeOH, THF, H_2O, 70 °C, 96%.

NMR Spectra

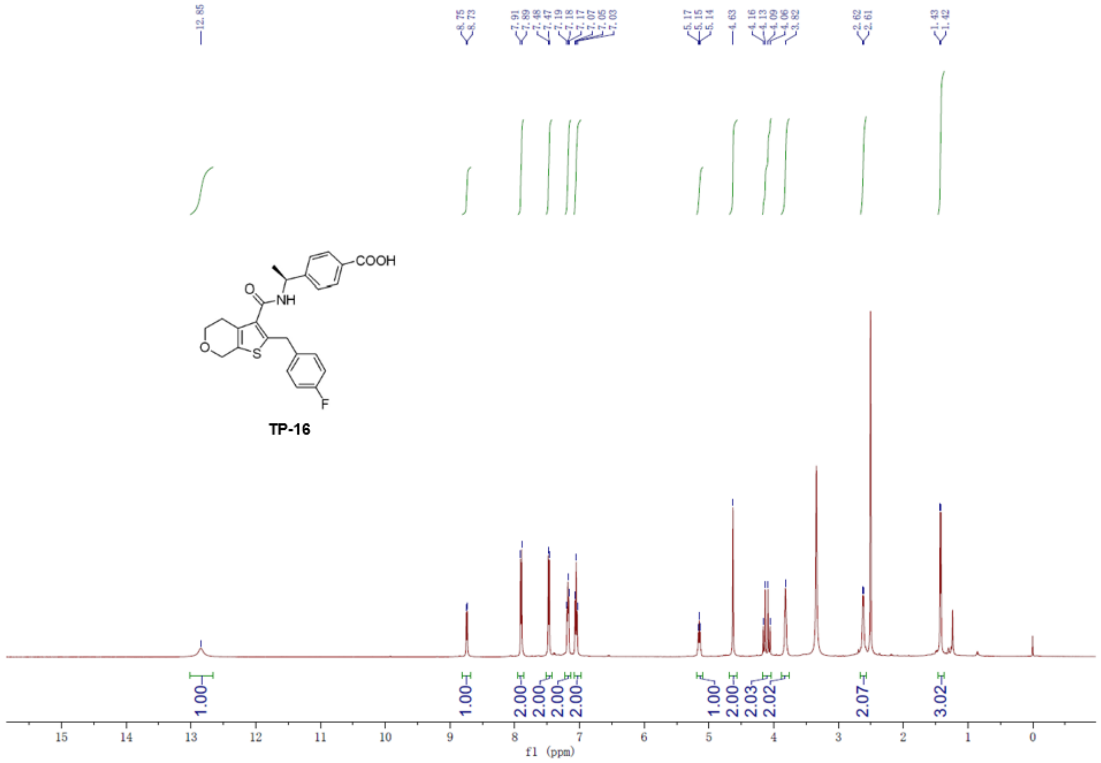
12.85
8.75
8.73
7.91
7.89
7.48
7.47
7.19
7.18
7.17
7.07
7.05
7.03
5.17
5.15
5.14
4.63
4.16
4.13
4.09
4.06
3.82
2.62
2.61
1.43
1.42
COOH
NH
O
S
F
TP-16
1.00
1.00
2.00
2.00
2.00
2.00
1.00
2.00
2.03
2.02
2.07
3.02
15 14 13 12 11 10 9 8 7 6 5 4 3 2 1 0
f1 (ppm)

Methods

Molecular Docking

Molecular docking studies were performed using Autodock4.2.6 (Morris et al., 2009). Ligand TP-16 was docked into the EP4 receptor bound to an antagonist, ONO-AE3-208 (PDB: 5YWY) (Toyoda et al., 2019). A grid of 60, 60, and 60 points in x, y, and z directions was created with a grid spacing of 0.375 Å at the binding site for docking. The program AutoDockTool-1.5.6 was used for preparing EP4 receptor and ligand TP-16 parameter files. A Lamarckian genetic algorithm was used for generating binding poses (Morris et al., 1998). We computed 200 docking runs for TP-16, with the rotation of all non-ring torsion angles. The lowest binding energy conformation was selected for further analysis.

Preliminary pharmacokinetic studies of TP-16 *in vivo*

TP-16 was administered intravenously or orally by gavage as a 1 or 10 mg/kg solution, respectively (dose volume, 5 or 10 mL/kg respectively; dose vehicle, 5% (v/v) dimethyl sulfoxide (DMSO), 10% solutol, and 85% β-cyclodextrin) to male CD1 mice (n = 3). Blood samples were collected at 0.08, 0.25, 0.5, 1, 2, 4, 8, and 24 hr after administration. After suitable sample preparation, the concentration of TP-16 in plasma samples was determined by the Agilent 1290 HPLC system, coupled with a 6460 triple-quadrupole mass spectrometer and an Agilent Jet Stream electrospray ionization (ESI) source (Agilent Technologies, USA). Pharmacokinetic parameters were calculated by WinNonlin software version 7.0 based on non-compartmental analysis (Pharsight Corporation, Mountain View, USA). Mean plasma concentration-time curves were plotted by GraphPad Prism 6.0 (GraphPad software Inc., CA, USA).

Toxicity Evolution of TP-16

The *in vivo* toxicity test of TP-16 was performed by Shanghai Medicilon Inc. Briefly, Sprague-Dawley (SD) rats were randomly divided into two groups, vehicle group and TP-16 (100 mg/kg) group, and each group have six animals (three males and three females). TP-16 was dissolved in 0.5% MC400 and orally administrated once daily for 14 days. Abnormal behaviors and mortality were observed every day and body weight was recorded every three days. Animals were sacrificed on day 15, and blood parameters and organ morphology were recorded.

Reference

Morris GM, Goodsell DS, Halliday RS, Huey R, Hart WE, Belew RK*, et al.* Automated docking using a Lamarckian genetic algorithm and an empirical binding free energy function. J. Comput. Chem., 1998, 19: 1639-1662.

Morris GM, Huey R, Lindstrom W, Sanner MF, Belew RK, Goodsell DS, *et al.*. AutoDock4 and AutoDockTools4: Automated docking with selective receptor flexibility. J. Comput. Chem., 2009, 30: 2785-2791.

Toyoda Y, Morimoto K, Suno R, Horita S, Yamashita K, Hirata K, *et al.* (2019). Ligand binding to human prostaglandin E receptor EP4 at the lipid-bilayer interface. Nat. Chem. Biol., 2019, 15: 18-26.

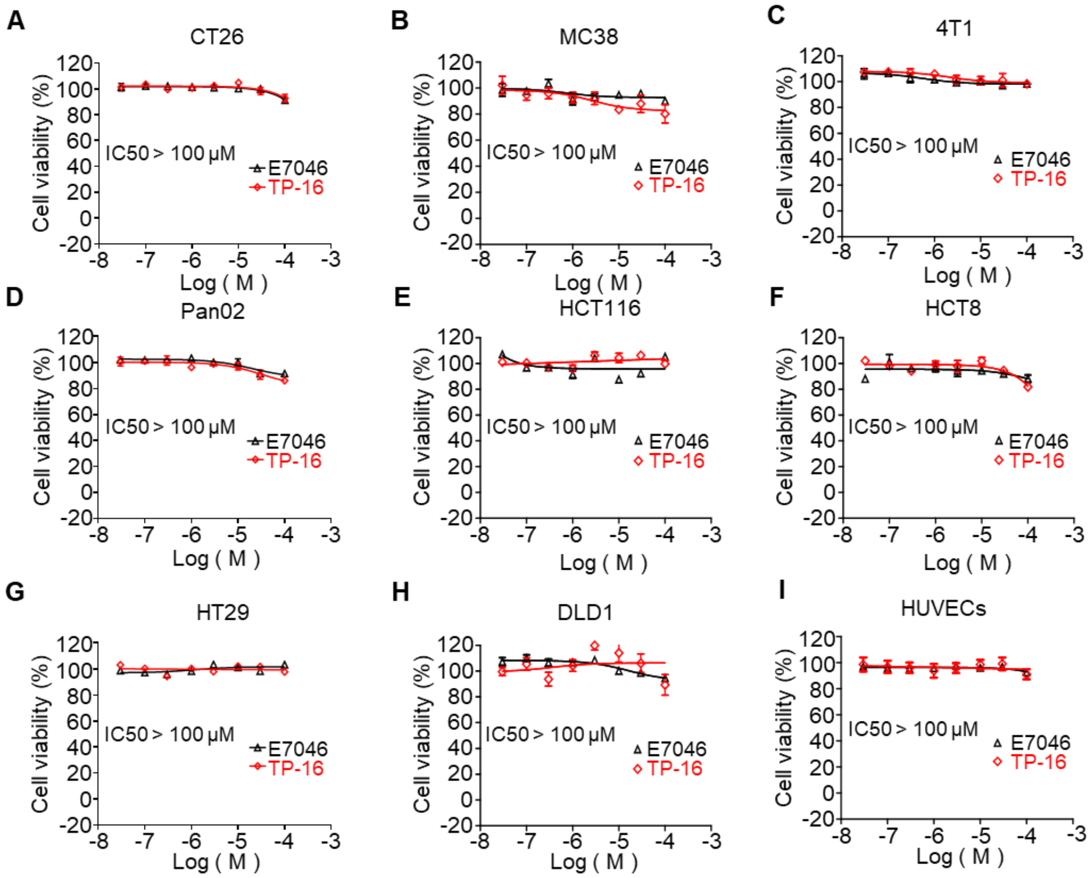

Appendix Figure S1. The *in vitro* cytotoxicity of TP-16. The cytotoxicity curves of E7046 and TP-16 against various cancer cells. Mouse cancer cells: CT26 (A), MC38 (B), 4T1 (C), Pan02 (D). Human colon cancer cells: HCT116 (E), HCT8 (F), HT29 (G) and DLD1 (H). Human normal cell line: HUVECs (I). Cell viability was measured by MTS. Data are presented as mean ± SEM derived from three independent experiments (n=3).

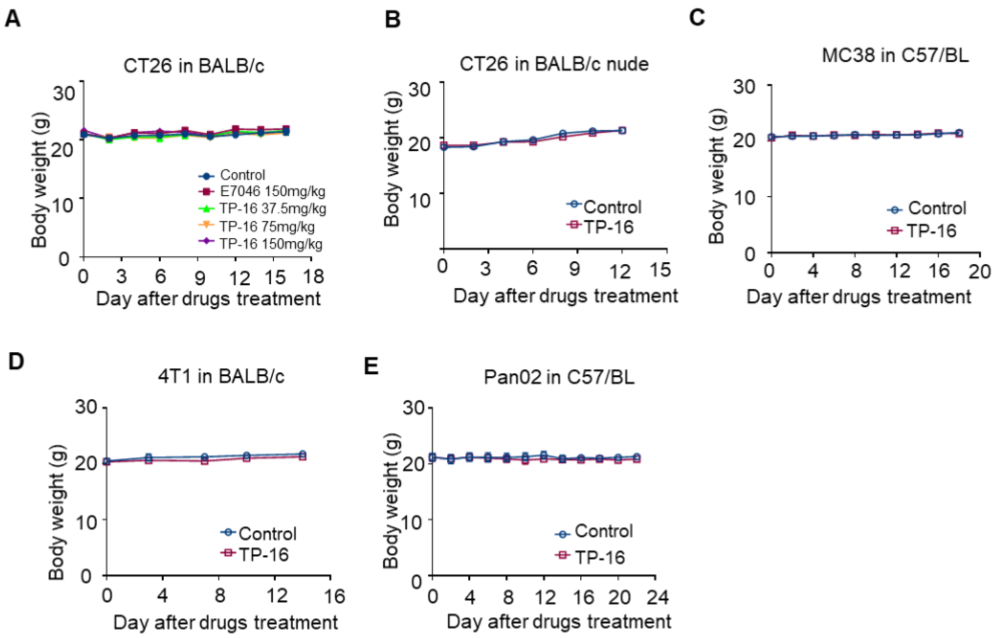

Appendix Figure S2. Mouse body weight of CT26 tumor-bearing BALB/c (A), CT26 tumor-bearing BALB/c nude mice (B), MC38 tumor-bearing C57/BL(C), 4T1 tumor-bearing BALB/c (D), and Pan02 tumor-bearing BALB/c (E) during indicated drug treatments.

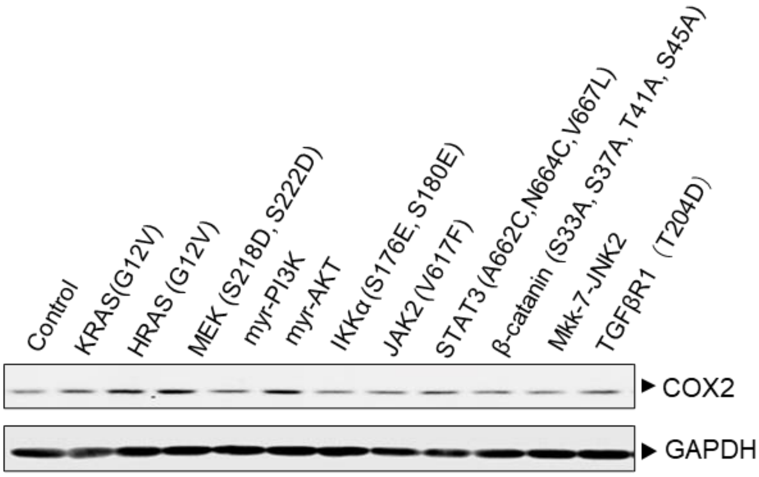

Appendix Figure S3. Multiple oncogenic events induce COX2 expression. CHO cells were transfected with indicated expression vector of mutated oncogenes for 24 hr, and then cells were harvested and lysed for the detection of COX2 expression by Western blot. GAPDH served as a loading control.

Appendix Table S1. Selectivity of TP-16 on GPCRs in LANCE Ultra cAMP assay.

Receptor	LANCE Ultra cAMP assay				
	Known agonists EC_{50} (μM)		Known antagonists IC_{50} (μM)		TP-16 IC_{50} (μM)
$AODRA_1$	NECA	0.0024	DPCPX	0.0032	>10[a]
$ADORA_{2A}$	NECA	0.00046	ZM-241385	0.0016	>10
$ADORA_{2B}$	NECA	0.0041	MRS 1754 hydrate	0.99	>10
$ADORA_3$	NECA	0.0040	MRS 1220	0.00074	>10
CB_1	CP55940	0.00053	AM251	0.0016	>10
CB_2	CP55940	0.00094	AM630	0.26	>10
DRD_2	Dopamine	0.0019	Spiperone	0.00027	>10
HTR_{1B}	RU24969	0.018	GR 55562 dihydrochloride	0.035	>10

[a]cAMP based GPCR selectivity assay was performed by Pharmaron (Beijing, China)

Appendix Table S2. Selectivity of TP-16 on GPCRs in calcium flux assay.

Receptor	Calcium flux assay				
	Known agonists EC_{50} (μM)		Known antagonists IC_{50} (μM)		TP-16 IC_{50} (μM)
ADRA1B	(R)-(-)-Phenylephrine hydrochloride	0.0014	Prazosin hydrochloride	0.0149	>10[a]
ADRA1D	Norepinephrine bitartrate monohydrate	0.0012	Prazosin hydrochloride	0.0046	>10
ADRA2A	UK 14,304 tartrate	0.0057	Rauwolscine hydrochloride	0.0169	>10
ADRB1	Isoprenaline hydrochloride	0.0055	CGP 20712 dihydrochloride	0.0185	>10
ADRB2	Isoprenaline hydrochloride	0.0111	ICI 118,551 hydrochloride	0.0021	>10
AT1	Angiotensin II 5-valine	0.0002	Irbesartan	0.0109	>10
AVPR1A	Oxytocin acetate	0.0087	Conivaptan hydrochloride	0.1325	>10
BB1	Neuromedin B	0.0002	PD 176252	0.8628	>10
CHRM1	Acetylcholine chloride	0.0014	Atropine sulfate monohydrate	0.0014	>10
CHRM2	Acetylcholine chloride	0.0040	Atropine sulfate monohydrate	0.0041	>10
CHRM3	Acetylcholine chloride	0.0003	Atropine sulfate monohydrate	0.0013	>10
DRD1	Dopamine hydrochloride	0.3038	SCH 23390 hydrochloride	0.0009	>10
HRH1	Histamine	0.0122	Mepyramine maleate	0.0059	>10
HTR2B	RU24969	0.0118	Ritanserin	0.0308	>10
OPRD1	ADL-5859	0.2663	Naltrindole hydrochloride	0.0012	>10
OPRK1	Nalfurafine hydrochloride	0.0029	Norbinaltorphimine dihydrochloride	0.0010	>10
OPRM1	Loperamide hydrochloride	0.0664	Alvimopan dihydrate	0.0023	>10

[a]Calcium flux based GPCR selectivity assay was performed by Pharmaron (Beijing, China).

Appendix Table S3. Pharmacokinetic parameters of TP-16 after oral administration or intravenous injection in CD1 mice.

Pharmacokinetic parameters	P.O. (10 mg/kg)	I.V. (1 mg/kg)
C_{max} (ng/mL)	3851.8	——
C_0 (ng/mL)	\	2370.2
$t_{1/2}$ (hr)	5.4	3.0
AUC_{0-24} (hr*ng/mL)	8399.8	2137.6
$AUC_{0-\infty}$ (hr*ng/mL)	8576.7	2140.6
V_d/F (mL/kg)	9083.1	2016.0
CL/F (mL/min/kg)	1166.0	467.2
MRT (hr)	3.7	2.1
Bioavailability (%)	40.1	——

Appendix Table S4. In vitro metabolic stability assessment using liver microsome.

Species	Remaining Percentages at 120 min (%)	In vitro T1/2 (min)	In vitro Clint (μL/min/10 6 cells)	Scale-up Clint (mL/min/kg)	Predicted hepatic Clint (mL/min/kg)	Hepatic Extraction Ratio (ER)
Human	90.5[a]	>581	<2.39	<6.07	<4.69	<0.227
Mouse	70.6	235	5.91	69.8	39.3	0.437

[a]Liver microsomal stability test was tested by WuXi AppTec (Shanghai, China)

Appendix Table S5. Summary of hematological data.

		Vehicle	TP-16 (100 mg/kg/day)
Male			
	WBC (10^9/L)	10.21 ± 1.26	11.81 ± 3.1
	RBC (10^{12}/L)	7.17 ± 0.01	6.92 ± 0.46
	HGB (g/L)	141 ± 0	137 ± 8
	HCT (%)	43.9 ± 0.8	43.1 ± 1.8
	MCV (fL)	61.3 ± 1.1	62.5 ± 1.6
	MCH (pg)	19.7 ± 0	19.8 ± 0.3
	MCHC (g/L)	322 ± 6	317 ± 5
	PLT (10^9/L)	1152 ± 26	868 ± 280
	Neutrophils (10^9/L)	0.88 ± 0.1	1.67 ± 0.49
	Neutrophils (%)	8.6 ± 0.1	15.6 ± 8.6
	Lymphocytes (10^9/L)	8.75 ± 1.22	9.48 ± 3.26
	Lymphocytes (%)	85.6 ± 1.5	79.1 ± 7.3
	Monocytes (10^9/L)	0.51 ± 0.04	0.57 ± 0.27
	Monocytes (%)	5.1 ± 1.1	4.7 ± 1.7
	Eosinophils (10^9/L)	0.06 ± 0.02	0.06 ± 0.02
	Eosinophils (%)	0.6 ± 0.3	0.5 ± 0.2
	Basophils (10^9/L)	0.02 ± 0	0.02 ± 0.01
	Basophils (%)	0.2 ± 0	0.2 ± 0.1
	Reticulocytes (%)	8.53 ± 0.83	9.14 ± 1.16
	Reticulocytes (10^9/L)	611.2 ± 60.4	629.4 ± 50.9
Female			
	WBC (10^9/L)	9.55 ± 1.81	10.81 ± 0.81
	RBC (10^{12}/L)	7.7 ± 0.36	8.08 ± 0.37
	HGB (g/L)	147 ± 7	146 ± 5
	HCT (%)	44.7 ± 1.9	45.1 ± 1
	MCV (fL)	58.1 ± 1.4	55.8 ± 1.5
	MCH (pg)	19.1 ± 0.3	18.1 ± 0.3
	MCHC (g/L)	328 ± 4	325 ± 4
	PLT (10^9/L)	1124 ± 82	1152 ± 95
	Neutrophils (10^9/L)	0.75 ± 0.09	1.21 ± 0.08
	Neutrophils (%)	8 ± 0.7	11.3 ± 1.4
	Lymphocytes (10^9/L)	8.24 ± 1.62	8.98 ± 0.68
	Lymphocytes (%)	86.1 ± 1.1	83.1 ± 1.2
	Monocytes (10^9/L)	0.45 ± 0.14	0.5 ± 0.17
	Monocytes (%)	4.6 ± 1	4.6 ± 1.3
	Eosinophils (10^9/L)	0.1 ± 0.02	0.1 ± 0.02

Eosinophils (%)	1.1 ± 0.4	0.9 ± 0.2
Basophils (10^9/L)	0.02 ± 0.01	0.01 ± 0.01
Basophils (%)	0.2 ± 0.1	0.13 ± 0.06
Reticulocytes (%)	5.68 ± 1.58	5.56 ± 0.68
Reticulocytes (10^9/L)	434.9 ± 109.9	448.2 ± 38.6

Data expressed as means ± SD. *Significantly different from control group ($p < 0.05$).

Appendix Table S6. Summary of organ data.

		Male		Female	
Organ		Vehicle	TP-16	Vehicle	TP-16
Liver	Normal	3/3	3/3	3/3	3/3
Kidney	Normal	3/3	3/3	3/3	3/3
Thyroid gland	Normal	3/3	3/3	3/3	3/3
Urinary bladder	Normal	3/3	3/3	3/3	3/3
Spleen	Normal	3/3	3/3	3/3	3/3
Pancreas	Normal	3/3	3/3	3/3	3/3
Thymus	Normal	3/3	3/3	3/3	3/3
Thyroid gland	Normal	3/3	3/3	3/3	3/3
Parathyroid gland	Normal	3/3	3/3	3/3	3/3
Trachea	Normal	3/3	3/3	3/3	3/3
Esophagus	Normal	3/3	3/3	3/3	3/3
Lung	Normal	3/3	3/3	3/3	3/3
Heart	Normal	3/3	3/3	3/3	3/3
Salivary gland	Normal	3/3	3/3	3/3	3/3
Cervical lymph node	Normal	3/3	3/3	3/3	3/3
Mesenteric lymph node	Normal	3/3	3/3	3/3	3/3
Stomach	Normal	3/3	3/3	3/3	3/3
Duodenum	Normal	3/3	3/3	3/3	3/3
Jejunum	Normal	3/3	3/3	3/3	3/3
Ileum	Normal	3/3	3/3	3/3	3/3
Colon	Normal	3/3	3/3	3/3	3/3
Rectum	Normal	3/3	3/3	3/3	3/3
Preputial/Clitoral gland	Normal	3/3	3/3	3/3	3/3
Skin/ Mammary gland	Normal	3/3	3/3	3/3	3/3
Eye	Normal	3/3	3/3	3/3	3/3
Harderian gland	Normal	3/3	3/3	3/3	3/3
Brain	Normal	3/3	3/3	3/3	3/3
Pituitary gland	Normal	3/3	3/3	3/3	3/3
Femur/Bone marrow	Normal	3/3	3/3	3/3	3/3
Nasal cavity	Normal	3/3	3/3	3/3	3/3
Testis	Normal	3/3	3/3	3/3	3/3
Epididymis	Normal	3/3	3/3	-	-

Prostate	Normal	3/3	3/3	-	-
Seminal vesicle	Normal	3/3	3/3	-	-
Ovary	Normal	-	-	3/3	3/3
Uterus	Normal	-	-	3/3	3/3
Vagina	Normal	-	-	3/3	3/3

Appendix Table S7. Primers for q-PCR

Gene	Origin	Forward	Reverse
Ptger1	Mouse	GGGATGCTCGAAACACCAGA	TTGGGGTTTTAAGGCCGTGT
Ptger2	Mouse	GGTCCTGAGGTTAATGCGCT	TGGCACTGGACTGGGTAGAA
Ptger3	Mouse	TGTCGGTTGAGCAATGCAAGACAC	TCTGGCAGAACTTCCGAAGAAGGA
Ptger4	Mouse	TTTCTTCGGTCTGTCGGGTC	GGCTGTAGAAGTAGGCGTGG
Cxcl10	Mouse	ATGACGGGCCAGTGAGAATG	GAGGCTCTCTGCTGTCCATC
Tnfa	Mouse	AGGCACTCCCCCAAAAGATG	CCACTTGGTGGTTTGTGAGTG
Ccl2	Mouse	TTAAAAACCTGGATCGGAACCAA	GCATTAGCTTCAGATTTACGGGT
Ccl5	Mouse	TGCTTTGCCTACCTCTCCCT	ACACACTTGGCGGTTCCTTC
Arg-1	Mouse	ACATTGGCTTGCGAGACGTA	ATCACCTTGCCAATCCCCAG
Ptgs2	Mouse	CATCCCCTTCCTGCGAAGTT	CATGGGAGTTGGGCAGTCAT
Il-4ra	Mouse	ACACCAATGTGTCCGACGAA	CTGCAGGGTTGTCCTCTCTG
Ido1	Mouse	CCCAGTCCGTGAGTTTGTCA	TCTTCCGACTTGTCGCCATC
Il-10	Mouse	AAGGGTTACTTGGGTTGCCA	GCCTGGGGCATCACTTCTAC
CD206	Mouse	CATGAGGCTTCTCCTGCTTCTG	TTGCCGTCTGAACTGAGATGG
Fizzl	Mouse	CCAATCCAGCTAACTATCCCTCC	CCAGTCAACGAGTAAGCACAG
Ym1	Mouse	AGAAGGGAGTTTCAAACCTGGT	CTCTTGCTGATGTGTGTAAGTGA
Il-6	Mouse	GGGACTGATGCTGGTGACAA	ACAGGTCTGTTGGGAGTGGT
Il-1β	Mouse	TGCCACCTTTTGACAGTGATG	AAGGTCCACGGGAAAGACAC
Cxcl1	Mouse	CCATCCAGAGCTTGACGGTG	TGGGGGTTGAGGCAAACTTC
β-Actin	Mouse	GTACGCCAACACAGTGCTG	CGTCATACTCCTGCTTGCTG

Appendix Table S8. P values and statistical tests in figures.

Figure 1	P value	Comparison	Statistical test
1A	0.079	EP2: bone marrow myeloid cells vs colon tumor myeloid cells	Kolmogorov–Smirnov test
	0.015	EP4: bone marrow myeloid cells vs colon tumor myeloid cells	Kolmogorov–Smirnov test
1E	0.0087	(% $F4/80^{+}CD11c^{-}$ Macrophage of BMMC) GM-CSF/IL-4 vs GM-CSF/IL-4 + PGE_2	Unpaired t test
	0.025	(% $F4/80^{+}CD11c^{-}$ Macrophage of BMMC) GM-CSF/IL-4 + PGE_2 vs GM-CSF/IL-4 + PGE_2 + E7046	Unpaired t test
	0.017	(% $F4/80^{-}CD11c^{+}$ DC of BMMC) GM-CSF/IL-4 vs GM-CSF/IL-4 + PGE_2	Unpaired t test
	0.0035	(% $F4/80^{-}CD11c^{+}$ DC of BMMC) GM-CSF/IL-4 vs GM-CSF/IL-4 + PGE_2 + E7046	Unpaired t test
1H	0.00090	(% mMDSC of BMMC) GM-CSF/IL-6 vs GM-CSF/IL-6 + PGE_2	Unpaired t test
	0.0011	(% mMDSC of BMMC) GM-CSF/IL-6 + PGE_2 vs GM-CSF/IL-6 + PGE_2 + PF-04418948	Unpaired t test
	0.0034	(% mMDSC of BMMC) GM-CSF/IL-6 + PGE_2 vs GM-CSF/IL-6 + PGE_2 + E7046	Unpaired t test
	0.00010	(% PMN-MDSC of BMMC) GM-CSF/IL-6 vs GM-CSF/IL-6 + PGE_2	Unpaired t test
	0.0012	(% PMN-MDSC of BMMC) GM-CSF/IL-6 + PGE_2 vs GM-CSF/IL-6 + PGE_2 +E7046	Unpaired t test
**Figure 3**	**P value**	**Comparison**	**Statistical test**
3B	0.0231	Control vs TP-16 75mg/kg	One-way ANOVA

	0.0221	Control vs TP-16 150mg/kg	One-way ANOVA
3C	0.33	Control vs TP-16	Unpaired t test
3D	0.0045	Control vs TP-16	Unpaired t test
3E	0.0055	Control vs TP-16	Unpaired t test
3F	0.0012	Control vs TP-16	Unpaired t test
3G	0.038	Control vs TP-16	Unpaired t test
**Figure 4**	**P value**	**Comparison**	**Statistical test**
4A	0.0024	Control vs TP-16	Unpaired t test
4B	0.0027	Control vs TP-16	Unpaired t test
4C	0.0012	Control vs TP-16	Unpaired t test
4D	0.29	Control vs TP-16 (*Cxcl10*)	Unpaired t test
	< 0.0001	Control vs TP-16 (*Tnfa*)	Unpaired t test
	0.0055	Control vs TP-16 (*Ccl2*)	Unpaired t test
	0.0086	Control vs TP-16 (*Ccl5*)	Unpaired t test
4E	0.044	Control vs TP-16 (*Arg-1*)	Unpaired t test
	0.0017	Control vs TP-16 (*CD206*)	Unpaired t test
	0.0089	Control vs TP-16 (*Fizzl*)	Unpaired t test
	0.00010	Control vs TP-16 (*Ym1*)	Unpaired t test
4F	0.033	Control vs TP-16 (*Arg-1*)	Unpaired t test
	0.0004	Control vs TP-16 (*Ptgs2*)	Unpaired t test
	0.0023	Control vs TP-16 (*Il-4ra*)	Unpaired t test
	0.044	Control vs TP-16 (*Ido1*)	Unpaired t test
	< 0.0001	Control vs TP-16 (*Il-10*)	Unpaired t test
4G	0.0018	Control vs TP-16 (*Arg-1*)	Unpaired t test
	0.0029	Control vs TP-16 (*Ptgs2*)	Unpaired t test
	0.026	Control vs TP-16 (*Il-4ra*)	Unpaired t test
	0.0025	Control vs TP-16 (*Ido1*)	Unpaired t test
	0.014	Control vs TP-16 (*Il-10*)	Unpaired t test
4H	< 0.0001	Control vs TP-16	Unpaired t test
4I	0.0012	Control vs TP-16	Unpaired t test
4J	0.0005	Control vs TP-16	Unpaired t test
4K	0.0015	Control vs TP-16	Unpaired t test
**Figure 5**	**P value**	**Comparison**	**Statistical test**
5A	< 0.0001	Control vs IFNγ (*Cxcl10*)	One-way ANOVA
	0.0044	Control + IFNγ vs Control + IFNγ + PGE_2 (*Cxcl10*)	One-way ANOVA
	< 0.0001	Control + IFNγ + PGE_2 vs Control + IFNγ + PGE_2 + TP-16 (*Cxcl10*)	One-way ANOVA
	0.004	Control vs IFNγ (*Tnfa*)	One-way ANOVA
	0.44	Control + IFNγ vs Control + IFNγ + PGE_2 (*Tnfa*)	One-way ANOVA
	< 0.0001	Control + IFNγ + PGE_2 vs	One-way ANOVA

		Control + IFNγ + PGE_2 + TP-16 (*Tnfa*)	
	0.058	Control vs IFNγ (*Arg-1*)	One-way ANOVA
	< 0.0001	Control + IFNγ vs Control + IFNγ + PGE_2 (*Arg-1)*	One-way ANOVA
	< 0.0001	Control + IFNγ + PGE_2 vs Control + IFNγ + PGE_2 + TP-16 (*Arg-1*)	One-way ANOVA
	< 0.0001	Control vs IFNγ (*Ym-1*)	One-way ANOVA
	0.001	Control + IFNγ vs Control + IFNγ + PGE_2 (*Ym-1)*	One-way ANOVA
	0.0004	Control + IFNγ + PGE_2 vs Control + IFNγ + PGE_2 + TP-16 (*Ym-1*)	One-way ANOVA
5B	< 0.0001	Control vs IL-4 (*Arg-1*)	One-way ANOVA
	< 0.0001	Control + IL-4 vs Control + IL-4 + PGE_2 (*Arg-1)*	One-way ANOVA
	< 0.0001	Control + IL-4 + PGE_2 vs Control + IL-4 + PGE_2 + TP-16 (*Arg-1*)	One-way ANOVA
	<0.0001	Control vs IL-4 (*Ym-1*)	One-way ANOVA
	0.034	Control + IL-4 vs Control + IL-4 + PGE_2 (*Ym-1)*	One-way ANOVA
	0.0003	Control + IL-4 + PGE_2 vs Control + IL-4 + PGE_2 + TP-16 (*Ym-1*)	One-way ANOVA
	<0.0001	Control vs IL-4 (*CD206*)	One-way ANOVA
	0.011	Control + IL-4 vs Control + IL-4 + PGE_2 (*CD206)*	One-way ANOVA
	0.013	Control + IL-4 + PGE_2 vs Control + IL-4 + PGE_2 + TP-16 (*CD206*)	One-way ANOVA
5C	0.007	(% mMDSC of BMMC) GM-CSF/IL-6 vs GM-CSF/IL-6 + PGE_2	One-way ANOVA
	0.048	(% mMDSC of BMMC) GM-CSF/IL-6 + PGE_2 vs GM-CSF/IL-6 + PGE_2 + 0.1 μM TP16	One-way ANOVA
	0.012	(% mMDSC of BMMC) GM-CSF/IL-6 + PGE_2 vs GM-CSF/IL-6 + PGE_2 + 1 μM TP16	One-way ANOVA

	0.008	(% mMDSC of BMMC) GM-CSF/IL-6 + PGE_2 vs GM-CSF/IL-6 + PGE_2 + 10 μM TP16	One-way ANOVA
	<0.0001	(% PMN-MDSC of BMMC) GM-CSF/IL-6 vs GM-CSF/IL-6 + PGE_2	One-way ANOVA
	0.0014	(% PMN-MDSC of BMMC) GM-CSF/IL-6 + PGE_2 vs GM-CSF/IL-6 + PGE_2 + 0.1 μM TP16	One-way ANOVA
	0.0006	(% PMN-MDSC of BMMC) GM-CSF/IL-6 + PGE_2 vs GM-CSF/IL-6 + PGE_2 + 1 μM TP16	One-way ANOVA
	<0.0001	(% PMN-MDSC of BMMC) GM-CSF/IL-6 + PGE_2 vs GM-CSF/IL-6 + PGE_2 + 10 μM TP16	One-way ANOVA
5D	<0.0001	GM-CSF/IL-6 vs GM-CSF/IL-6 + PGE_2 (*Arg-1*)	One-way ANOVA
	0.0001	GM-CSF/IL-6 + PGE_2 vs GM-CSF/IL-6 + PGE_2 + TP16 (*Arg-1*)	One-way ANOVA
	<0.0001	GM-CSF/IL-6 vs GM-CSF/IL-6 + PGE_2 (*Ptgs2*)	One-way ANOVA
	0.0006	GM-CSF/IL-6 + PGE_2 vs GM-CSF/IL-6 + PGE_2 + TP16 (*Ptgs2*)	One-way ANOVA
	0.040	GM-CSF/IL-6 vs GM-CSF/IL-6 + PGE_2 (*Il-10*)	One-way ANOVA
	0.010	GM-CSF/IL-6 + PGE_2 vs GM-CSF/IL-6 + PGE_2 + TP16 (*Il-10*)	One-way ANOVA
	0.030	GM-CSF/IL-6 vs GM-CSF/IL-6 + PGE_2 (*Il-4ra*)	One-way ANOVA
	0.22	GM-CSF/IL-6 + PGE_2 vs GM-CSF/IL-6 + PGE_2 + TP16 (*Il-4ra*)	One-way ANOVA
5F	<0.0001	Naïve $CD8^+$ vs Activated $CD8^+$	One-way ANOVA
	<0.0001	Activated $CD8^+$ vs Activated	One-way ANOVA

		$CD8^+$ & Control Tumor $CD11b^+$ (2:1)	
	<0.0001	Activated $CD8^+$ vs Activated $CD8^+$ & Control Tumor $CD11b^+$ (1:1)	One-way ANOVA
	<0.0001	Activated $CD8^+$ vs Activated $CD8^+$ & Control Tumor $CD11b^+$ (1:2)	One-way ANOVA
	<0.0001	Activated $CD8^+$ & Control Tumor $CD11b^+$ vs Activated $CD8^+$ & TP-16 Tumor $CD11b^+$ (2:1)	One-way ANOVA
	<0.0001	Activated $CD8^+$ & Control Tumor $CD11b^+$ vs Activated $CD8^+$ & TP-16 Tumor $CD11b^+$ (1:1)	One-way ANOVA
	<0.0001	Activated $CD8^+$ & Control Tumor $CD11b^+$ vs Activated $CD8^+$ & TP-16 Tumor $CD11b^+$ (1:2)	One-way ANOVA
**Figure 6**	**P value**	**Comparison**	**Statistical test**
6A	0.0058	Control vs TP-16	One-way ANOVA
	<0.0001	Control vs anti-PD-1	One-way ANOVA
	<0.0001	Control vs combination	One-way ANOVA
6B	0.0066	Control vs TP-16	Log-rank test
	0.0008	Control vs anti-PD-1	Log-rank test
	<0.0001	Control vs combination	Log-rank test
	0.015	TP-16 vs combination	Log-rank test
**Figure 7**	**P value**	**Comparison**	**Statistical test**
7C	0.012	Control vs TP-16	One-way ANOVA
	0.0091	Control vs anti-PD-1	One-way ANOVA
	0.0005	Control vs combination	One-way ANOVA
	0.039	Control vs anti-PD-1 (0-2 mm)	Unpaired t test
	0.0019	Control vs combination (2-4 mm)	Unpaired t test
	0.012	Control vs TP-16	Unpaired t test
	0.0006	anti-PD-1 vs TP-16	Unpaired t test
	0.0038	Control vs combination (2-4 mm)	Unpaired t test
**Figure EV2**	**P value**	**Comparison**	**Statistical test**
EV2A	0.0024	Control vs celecoxib	One-way ANOVA

	0.0004	Control vs TP-16	One-way ANOVA
EV2B	<0.0001	Control vs TP-16	Unpaired t test
EV2C	0.0061	Control vs TP-16	Unpaired t test
EV2D	0.040	Control vs TP-16	Unpaired t test
EV2E	0.0089	Control vs TP-16	Unpaired t test
EV2F	0.0007	Control vs TP-16	Unpaired t test
EV2H	0.018	Control vs TP-16 (M1)	Unpaired t test
	0.0042	Control vs TP-16 (M2)	Unpaired t test
	0.018	Control vs TP-16 (mMDSC)	Unpaired t test
	0.0067	Control vs TP-16 (PMN-MDSC)	Unpaired t test
EV2I	<0.0001	Control vs TP-16	Unpaired t test
EV2J	0.0004	Control vs TP-16	Unpaired t test
**Figure EV3**	**P value**	**Comparison**	**Statistical test**
EV3A	<0.0001	M-CSF + IL-4 vs M-CSF + IL-4 + PGE_2	One-way ANOVA
	<0.0001	M-CSF + IL-4 + PGE_2 vs M-CSF + IL-4 + PGE_2 + 10 μM TP-16 (Si)	One-way ANOVA
	<0.0001	M-CSF + IL-4 + PGE_2 vs M-CSF + IL-4 + PGE_2 + 1 μM TP-16 (Si)	One-way ANOVA
	<0.0001	M-CSF + IL-4 + PGE_2 vs M-CSF + IL-4 + PGE_2 + 0.1 μM TP-16 (Si)	One-way ANOVA
	<0.0001	M-CSF + IL-4 + PGE_2 vs M-CSF + IL-4 + PGE_2 + 10 μM TP-16 (Se)	One-way ANOVA
	<0.0001	M-CSF + IL-4 + PGE_2 vs M-CSF + IL-4 + PGE_2 + 1 μM TP-16 (Se)	One-way ANOVA
	<0.0001	M-CSF + IL-4 + PGE_2 vs M-CSF + IL-4 + PGE_2 + 0.1 μM TP-16 (Se)	One-way ANOVA
EV3E	<0.0001	Naïve $CD4^+$ vs Activated $CD4^+$	One-way ANOVA
	<0.0001	Activated $CD4^+$ vs Activated $CD4^+$ & Control Tumor $CD11b^+$ (2:1)	One-way ANOVA
	<0.0001	Activated $CD4^+$ vs Activated $CD4^+$ & Control Tumor	One-way ANOVA

		$CD11b^+$ (1:1)	
	<0.0001	Activated $CD4^+$ vs Activated $CD4^+$ & Control Tumor $CD11b^+$ (1:2)	One-way ANOVA
	0.076	Activated $CD4^+$ & Control Tumor $CD11b^+$ vs Activated $CD4^+$ & TP-16 Tumor $CD11b^+$ (2:1	One-way ANOVA
	<0.0001	Activated $CD4^+$ & Control Tumor $CD11b^+$ vs Activated $CD4^+$ & TP-16 Tumor $CD11b^+$ (1:1)	One-way ANOVA
	<0.0001	Activated $CD4^+$ & Control Tumor $CD11b^+$ vs Activated $CD4^+$ & TP-16 Tumor $CD11b^+$ (1:2)	One-way ANOVA
**Figure EV4**	**P value**	**Comparison**	**Statistical test**
EV4B	0.016	Control vs TP-16	One-way ANOVA
	0.0056	Control vs anti-PD-1	One-way ANOVA
	<0.0001	Control vs combination	One-way ANOVA
EV4E	0.010	Control vs TP-16 (IL-6)	One-way ANOVA
	0.0001	Control vs anti-PD-1 (IL-6)	One-way ANOVA
	<0.0001	Control vs combination (IL-6)	One-way ANOVA
	0.0005	Control vs TP-16 (CXCL-1)	One-way ANOVA
	0.0001	Control vs anti-PD-1 (CXCL-1)	One-way ANOVA
	<0.0001	Control vs combination (CXCL1)	One-way ANOVA

Appendix Table S9. KEY RESOURCES TABLE

REAGENT or RESOURCE	SOURCE	IDENTIFIER
Antibodies		
PerCP/Cy5.5 anti-mouse CD45 antibody	BioLegend	103132
APC anti-mouse CD8a antibody	BioLegend	100712
PE anti-mouse IFN-γ antibody	BD Pharmingen	562020
PE anti-mouse Granzyme B antibody	eBioscience	12-8898-80
PE anti-mouse TNF-α antibody	BioLegend	506305
APC anti-mouse F4/80 antibody	BioLegend	123116
PE anti-mouse CD11c antibody	BioLegend	177307
APC anti-mouse CD11c antibody	BioLegend	117310
PE anti-mouse Ly6C antibody	BioLegend	128008
PerCP/Cy5.5 anti-mouse Ly6G antibody	BioLegend	127616
APC anti-mouse LY6G antibody	BioLegend	108412
FITC anti-mouse/human CD11b antibody	BioLegend	101206
APC anti-mouse CD4 antibody	BioLegend	100411
PerCP/Cy5.5 anti-mouse CD4 antibody	BioLegend	100433
PE anti-mouse MHC-II antibody	BioLegend	107608
PE anti-mouse CD206 antibody	BioLegend	141706
PE anti-mouse PD-1 antibody	BioLegend	135205
TruStain fcX	BioLegend	101320
LEAF™ Purified anti-mouse CD3 antibody	BioLegend	100207
LEAF™ Purified anti-mouse CD28 antibody	BioLegend	102111
In Vivo mAb anti-mouse	BioxCell	BE0273

PD-1		
Anti-Granzyme B antibody	Abcam	ab4059
Anti-CD8 antibody	Abcam	ab217344
Anti-Gr1 antibody	R&D	Mab1037
Anti-CD11b antibody	Abcam	Ab133357
Anti-PD-1 antibody	Servicebio	GB13338
Anti-PD-L1 antibody	Servicebio	GB13339
P-STAT3^{Tyr705} antibody	CST	9145
STAT3 antibody	CST	9139
P-AKT^{Ser473} antibody	CST	4060
AKT antibody	CST	9272
Arg1 antibody	Proteintech	16001
COX2 antibody	CST	12282
GAPDH antibody	Abcam	Ab181602
P-ERK$^{Thr218/Tyr220}$ antibody	CST	4370
ERK antibody	CST	9102
CD206 antibody	Abcam	ab64693
F4/80 antibody	Abcam	ab6640
P-CREB^{S133} antibody	Abcam	ab32096
TNFα antibody	Abcolonal	A11534
Chemicals, Peptides, and Recombinant Proteins		
Celecoxib	Sigma	PHR1683
PGE_2	Cayman Chemical	14010
ONO-8711	Cayman Chemical	14070
PF-04418948	TopScience	T3306
L-798106	Sigma	L4545
CFSE	Thermo	C34554
Recombinant murine GM-CSF	PeproTech	315-03
Recombinant murine IL-4	PeproTech	214-14
Recombinant murine IL-6	PeproTech	216-16
Recombinant murine M-CSF	PeproTech	315-02
Recombinant murine IFN-γ	PeproTech	315-05
Leukocyte Activation Cocktail, with BD GolgiPlug	BD Pharmingen	550583
Critical Commercial Assays		
Prostaglandin E2 Express EIA Kit	Cayman Chemical	500141
Mouse IL-6 ELISA KIT	Biolegend	431302
Mouse CXCL1 ELISA KIT	Abnova	KA0553

FLIPR Calcium 5 Assay Kit	Molecular Devices	R8185
GloSensor™ cAMP Assay kit	Promega	E1291
Luciferase Reporter Assay System	Promega	E1960
MTS Cell Titer 96 Cell Proliferation Assay	Promega	G3581
MojoSort™ Mouse CD3 T Cell Isolation Kit	BioLegend	480024
CD11b MicroBeads, human and mouse	Miltenyi	130-049-601
Fixation/Permeabillzation Solution Kit	BD Pharmingen	554714
Deposited DATA		
GSE23502	Yang XD et al., 2011	https://www.ncbi.nlm.nih.gov/gds.
GSE132004	This paper	
Experimental Models: Cell lines		
4T1	ATCC	CRL-2539
B16F10	CRCPUMC	TCM36
Pan02	CRCPUMC	N/A
MC38	CRCPUMC	N/A
CT26	ATCC	CRL-2638
HCT116	ATCC	CCL-247
HCT8	ATCC	CCL-244
HT29	ATCC	HTB-38
DLD1	ATCC	CCL-221
CHO	CRCPUMC	GNHa 3
CHO-Gα16	This paper	N/A
HEK293	CRCPUMC	GNHu43
HEK293T	CRCPUMC	GNHu17
HUVECs	This paper	N/A
Experimental Models: Organisms/Strains		
Mouse: BALB/c	National Rodent Laboratory Animal Center	N/A
Mouse: BALB/c nude	National Rodent Laboratory Animal	N/A

	Center	
Mouse: C57BL	National Rodent Laboratory Animal Center	N/A
Oligonucleotides		
PCR primers	q-PCR	See Appendix Table S1
Recombinant DNA		
Plasmid: LentiCRISPv2	Addgene	52961
Plasmid: psPAX2	Addgene	12260
Plasmid: pVSVg	Addgene	8454
Plasmid: Human Ptger1	cDNA Resource Center	PER0100000
Plasmid: Human Ptger2	cDNA Resource Center	PER020TN00
Plasmid: Human Ptger3	cDNA Resource Center	PER3VI0000
Plasmid: Human Ptger4	cDNA Resource Center	PER0400000
Plasmid: pCMV-Entry-Ptger4 (Mouse)	This paper	N/A
Plasmid: pcDNA3.1-3*Flag-Ptger4 (Rat)	This paper	N/A
Plasmid: pcDNA3.1-3*Flag- Ptger4 (Dog)	This paper	N/A
Plasmid: pcDNA3.1-3*Flag- Ptger4 (Monkey)	This paper	N/A
Plasmid: pGloSensor™-22F cAMP	Promega	E2301
Plasmid: CRE-luc	This paper	N/A
Plasmid: Tango-Ptger4	Addgene	66486
Plasmid: Kras (G12V)	Addgene	64602
Plasmid: Hras1 (G12V)	Addgene	64603
Plasmid: MEK1 (S218D,	Addgene	64604

S222D)		
Plasmid: PI3KCA	Addgene	64605
Plasmid: AKT1	Addgene	64606
Plasmid: IKKα (S176E, S180E)	Addgene	64607
Plasmid: JAK2 (V617F)	Addgene	64609
Plasmid: STAT3 (A662C, N664C, V617F)	Addgene	64610
Plasmid: β-catenin (S33A, S37A, T41A, S45A)	Addgene	64611
Plasmid: Mkk7-JNK2 fusion	Addgene	64618
Plasmid: TGFβR1 (T204D)	Addgene	64629
Software and Algorithms		
Flow Jo VX	Flow Jo, LLC	N/A
GraphPad Prism 7	GraphPad	N/A
