# Supplementary material for: Reprogramming immunosuppressive myeloid cells facilitates immunotherapy for colorectal cancer
Source: EMBO Mol Med. 2020 Dec 7;13(1):e12798. doi: 10.15252/emmm.202012798 (PMC7799360; doi:10.15252/emmm.202012798)
Supplement: Supplementary file 2 — Expanded View Figures PDF [file EMMM-13-e12798-s002.pdf]

Expanded View Figures

Figure EV1. Antagonistic activity, pharmacokinetic profiles, and safety of TP-16.

A Dose–response of TP-16 in cAMP reporter gene assay in HEK293 cells. Data are presented as mean ± SEM derived from three independent experiments (n = 3).

B Dose–effect curves of TP-16 in PGE_2-induced β-arrestin Tango assay. Data are presented as mean ± SEM derived from three independent experiments (n = 3).

C–E The antagonistic activities of TP-16 on EP4 were determined by a calcium flux assay by overexpressing Gα16 protein in CHO cells. Dose–response curves of TP-16 against EP4: monkey (C), rat (D), and mouse (E). Data are presented as mean ± SEM derived from three independent experiments (n = 3).

F, G The plasma concentration over time profiles of TP-16 after intravenous and oral administration of TP-16 at a dose of 1 and 10 mg/kg, respectively. Results are presented as mean ± SEM of three CD1 mice (n = 3).

H Body weights of male and female SD rats orally administered with TP-16 (100 mg/kg/day) in 14-day repeat-dose. Data are presented as means ± SD (n = 3/sex/group).

Data information: Data are presented as mean ± SEM except for H.

© 2021 The Authors

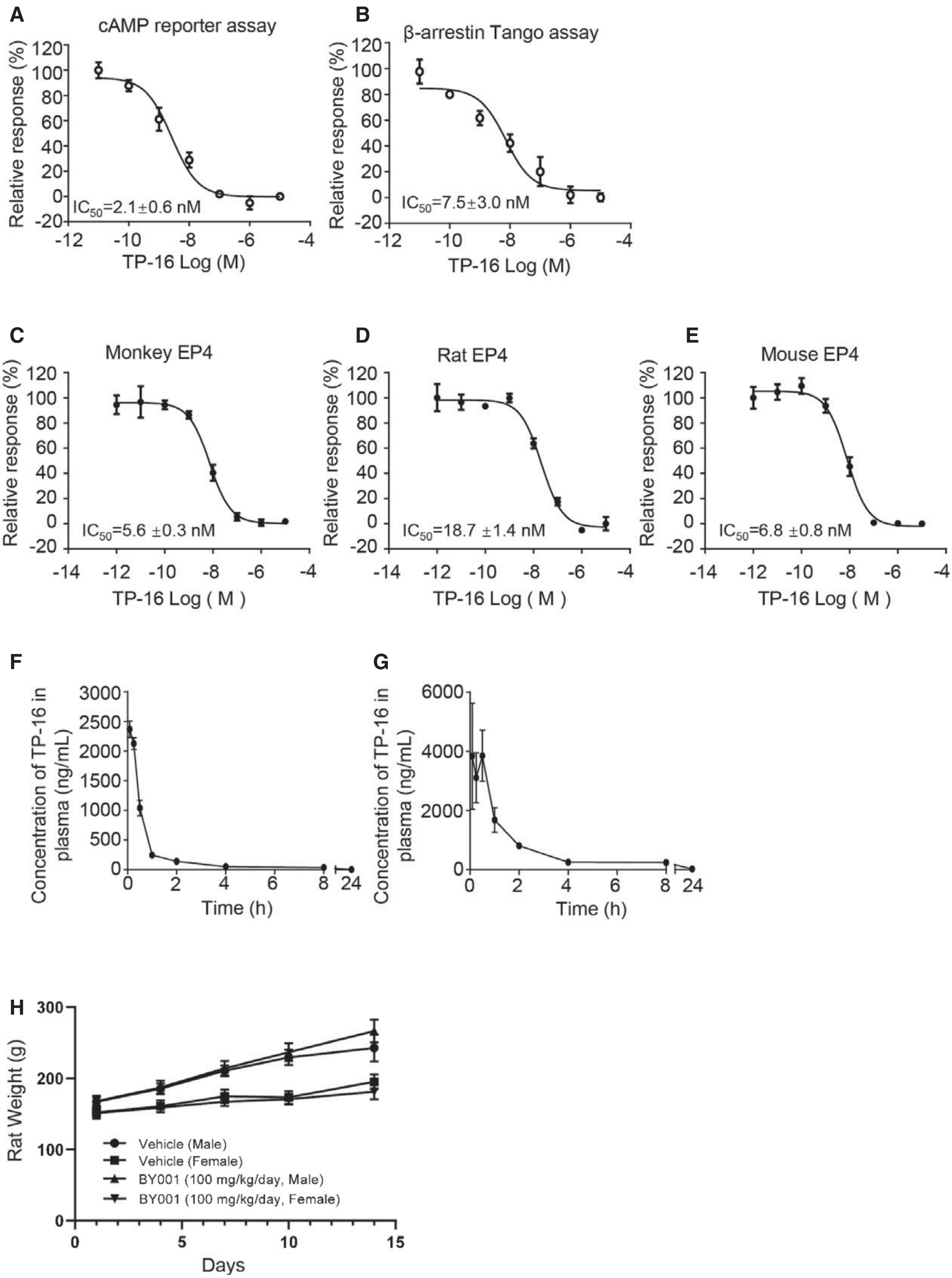

Figure EV1.

 © 2021 The Authors

Figure EV2. Effect of EP4 inhibition by TP-16 on tumor growth and subsets of intratumoral immune cells.

A The growth curves of CT26 xenograft tumors in BALB/c mice. CT26 tumor-bearing mice were treated with TP-16 (75 mg/kg, p.o., daily) or celecoxib (100 mg/kg, p.o., daily) for 16 days when tumor volumes reached 100-200 mm^3 (day 7). Data are presented as mean ± SEM. One-way analysis of variance (ANOVA), Tukey's multiple comparison; $***P < 0.001$. $n = 6$.

B, C The anti-tumor activity of TP-16 on 4T1 xenograft tumors in BALB/c mice and Pan02 xenograft tumors in C57BL/6 mice. Mice were treated with TP-16 (75 mg/kg, po, daily) when tumor volumes reached 100–200 mm^3 (day 7). Data are presented as mean ± SEM. A two-tailed unpaired Student's *t*-test was performed; $**P < 0.01$; $***P < 0.001$ ($n = 8$).

D, E Single-cell suspensions of tumors from CT26-bearing BALB/c mice treated with vehicle or 75 mg/kg TP-16 for 2 weeks were analyzed for intratumoral immune cells by flow cytometry analysis. Representative graphs and quantification of $CD11b^+F4/80^+$ macrophages (D) and $CD45^+CD11b^+CD11c^+MHC\ II^+$ dendritic cells (DCs) (E). Data are presented as mean ± SEM. A two-tailed unpaired Student's *t*-test was performed; $*P < 0.05$; $**P < 0.01$ ($n = 5$).

F Representative graphs and quantification of TNF-α in $CD11b^+$ cells isolated from the CT26 xenograft tumors ($n = 6$). Data are presented as mean ± SEM. A two-tailed unpaired Student's *t*-test was performed, $***P < 0.001$ versus the vehicle control group.

G Representative immunofluorescence images of p-CREB in $CD11b^+$ cells of CT26 tumor tissue. Scale bars, 50 μm.

H The changes of myeloid cell compartment in an orthotopic colorectal cancer mouse model, CT26-Luc model ($n = 4$). A two-tailed unpaired Student's *t*-test was performed, $*P < 0.05$, $**P < 0.01$ versus vehicle control group.

I, J Representative graphs and quantification of $CD4^+$ T-cell activation markers IFN-γ (I) and TNF-α (J) ($n = 5$). Data are presented as mean ± SEM derived from three independent experiments. A two-tailed unpaired Student's t-test was performed, $***P < 0.001$ versus the vehicle control group.

Source data are available online for this figure.

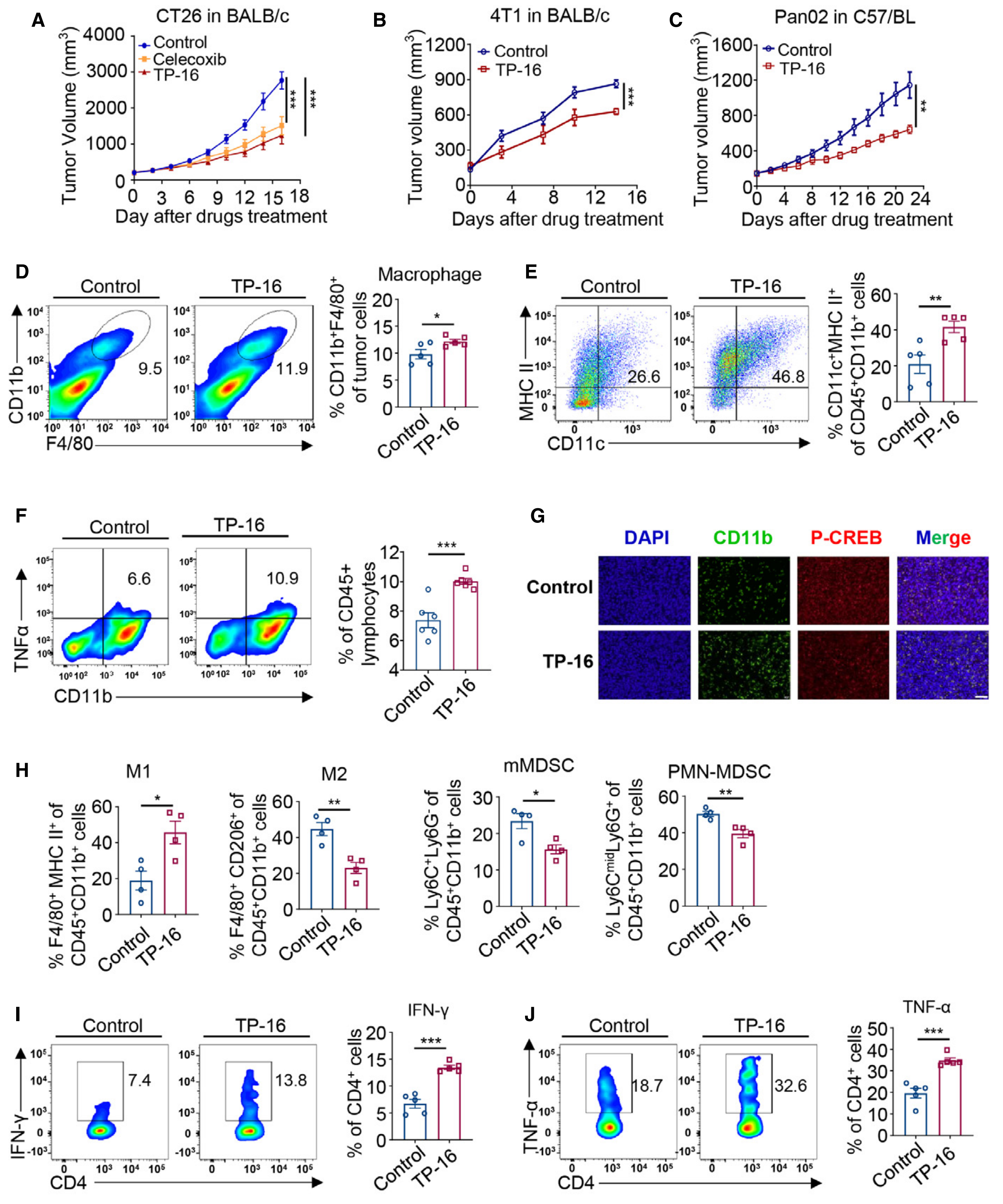

Figure EV2.

 © 2021 The Authors

Figure EV3. TP-16 suppresses M2 macrophage differentiation and reverses T-cell proliferation when cultured with tumor-infiltrating CD11b$^+$ myeloid cells.

A Mouse bone marrow cells were cultured with M-CSF/IL-4/PGE$_2$ for 6 days and varied concentrations of TP-16 was simultaneously (day 0) or sequentially (day 3) added. The generation of M2 macrophages (F4/80$^+$CD206$^+$) was analyzed by flow cytometry analysis. Si: simultaneous TP-16 treatment; Se: sequential TP-16 treatment. Data are presented as mean ± SEM. One-way analysis of variance (ANOVA) and Tukey's multiple comparison test were performed; ***$P < 0.001$ ($n = 3$).

B, C Mouse bone marrow-derived macrophages (BMMs) were stimulated with 20 ng/ml mouse recombinant IL-4 alone or in combination with 100 nM PGE$_2$, in the absence or presence of 10 μM TP-16 for the indicated time. The expression of p-STAT3^{705}, p-STAT3^{727}, p-STAT3, p-AKT^{473}, p-AKT^{308}, and AKT was detected by Western blotting. GAPDH served as the loading control.

D, E Representative histograms (D) and percentage (E) of CD4$^+$ T-cell proliferation at a ratio of 2:1, 1:1, and 1:2 CD4$^+$ T cells to CD11b$^+$ myeloid cells collected from CT26 tumor-bearing mice at 2 weeks post-treatment with vehicle or 75 mg/kg TP-16. All data are presented as mean ± SEM derived from three independent experiments ($n = 3$). A two-tailed unpaired Student's t-test was performed, ***$P < 0.001$.

Source data are available online for this figure.

© 2021 The Authors

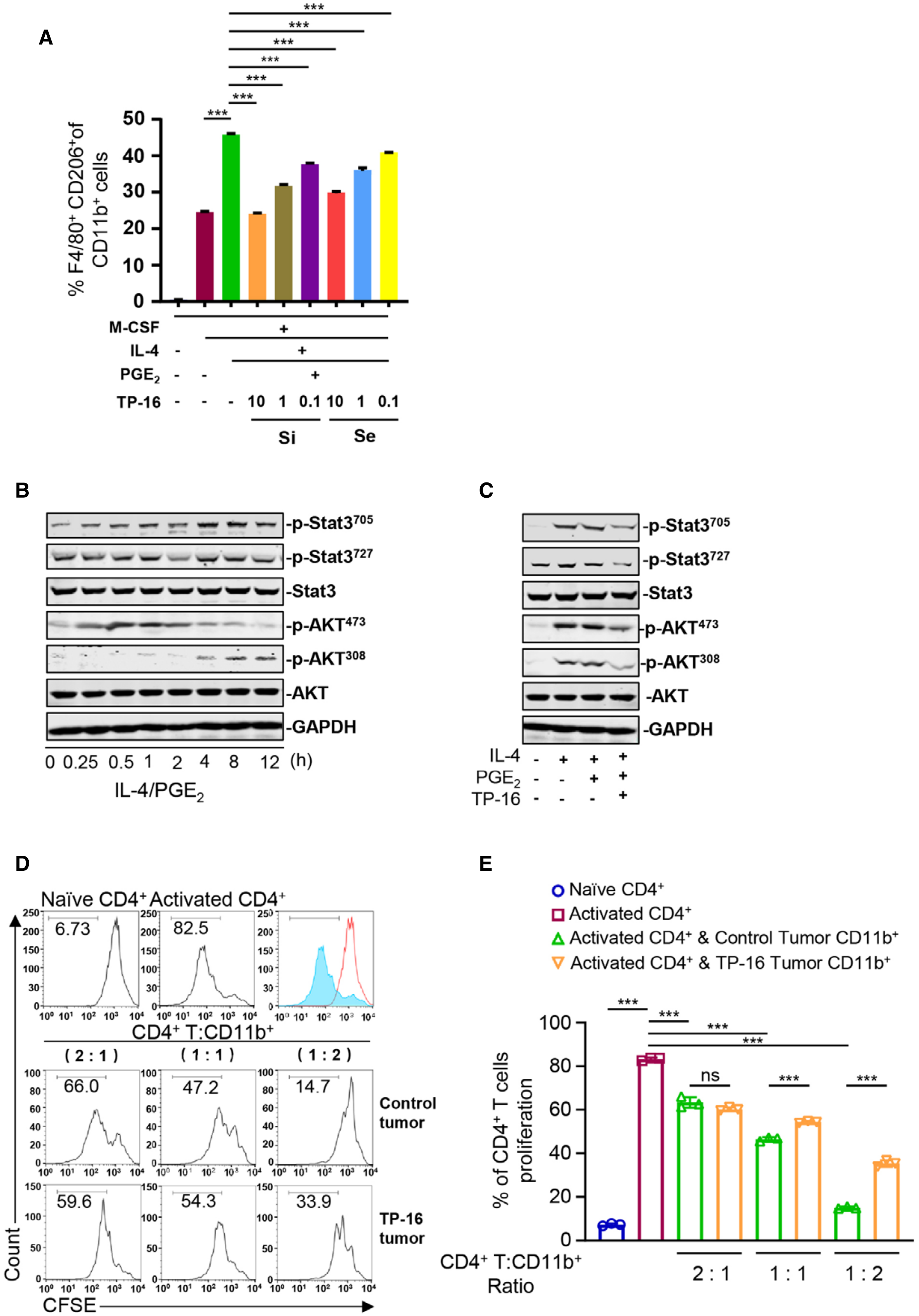

Figure EV3.

© 2021 The Authors

Figure EV4. In vivo efficacy of EP4 antagonist (TP-16) combined with immune checkpoint blockade.

A Body weight of mice (CT26 syngeneic tumor model).
B The growth of MC38 tumors treated with vehicle, TP-16 (75 mg/kg, po, daily), anti-PD-1 (50 μg, ip, twice weekly), and TP-16 combined with anti-PD-1 antibody (n = 8 per group). Data are presented as mean ± SEM. One-way ANOVA and Tukey's multiple comparison test were performed; *$P < 0.05$; **$P < 0.01$; ***$P < 0.001$.
C Body weight of mice (MC38 syngeneic tumor model).
D Representative immunofluorescence staining images of tumor sections from CT26 tumor-bearing mice stained for myeloid-derived suppressor cells (MDSCs; $CD11b^+Gr1^+$, upper panel) and M2 macrophages ($CD11b^+CD206^+$, lower panel). Scale bars, 50 μm.
E Cytokines (IL-6 and CXCL1) in the peripheral blood of CT26 tumor-bearing mice treated as indicated were measured by enzyme-linked immunosorbent assay (ELISA) on the last day of treatment (n = 6). One-way analysis of variance (ANOVA) and Turkey *post hoc* test were performed, *$P < 0.05$, **$P < 0.01$; ***$P < 0.001$.
F Pathway enrichment was analyzed based on the subsets of differentially expressed genes influenced by the combination therapy of TP-16 and anti-PD-1 antibody.

Source data are available online for this figure.

© 2021 The Authors

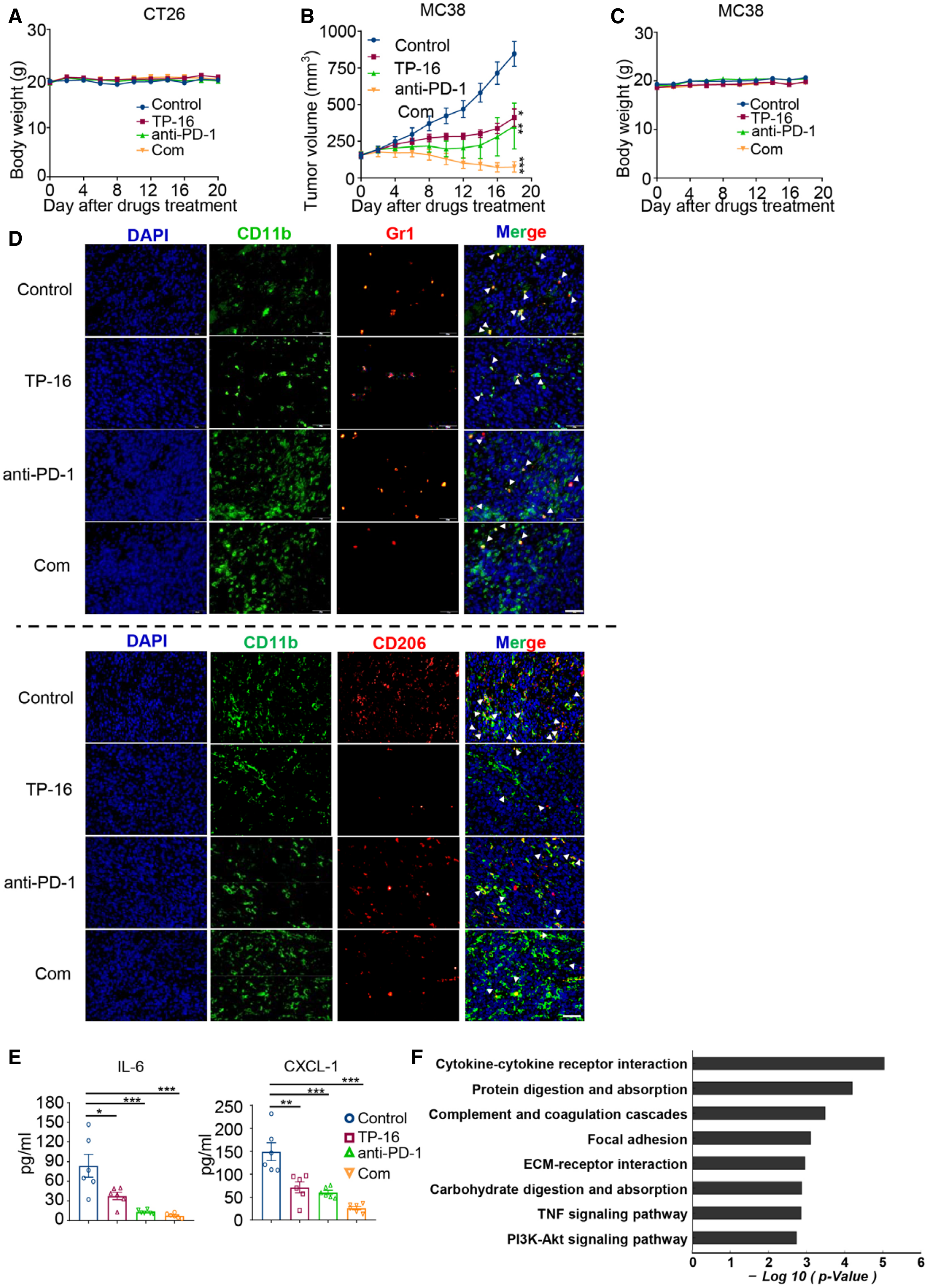

Figure EV4.

 © 2021 The Authors
