# Supplementary figures and images for: Reprogramming immunosuppressive myeloid cells facilitates immunotherapy for colorectal cancer
Source: EMBO Mol Med. 2020 Dec 7;13(1):e12798. doi: 10.15252/emmm.202012798 (PMC7799360; doi:10.15252/emmm.202012798)

### Appendix Figure S3

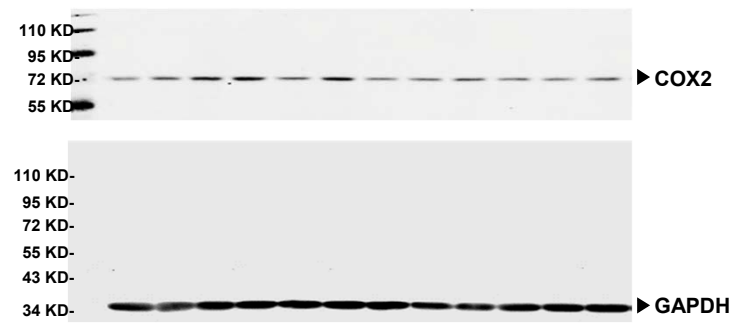

Supplement: Supplementary file 3 — Source Data for Expanded View and Appendix [file EMMM-13-e12798-s006.zip › EMM-2020-12798-V4-Appendix_Figure_source_data-sd.pdf]

Figure 3G

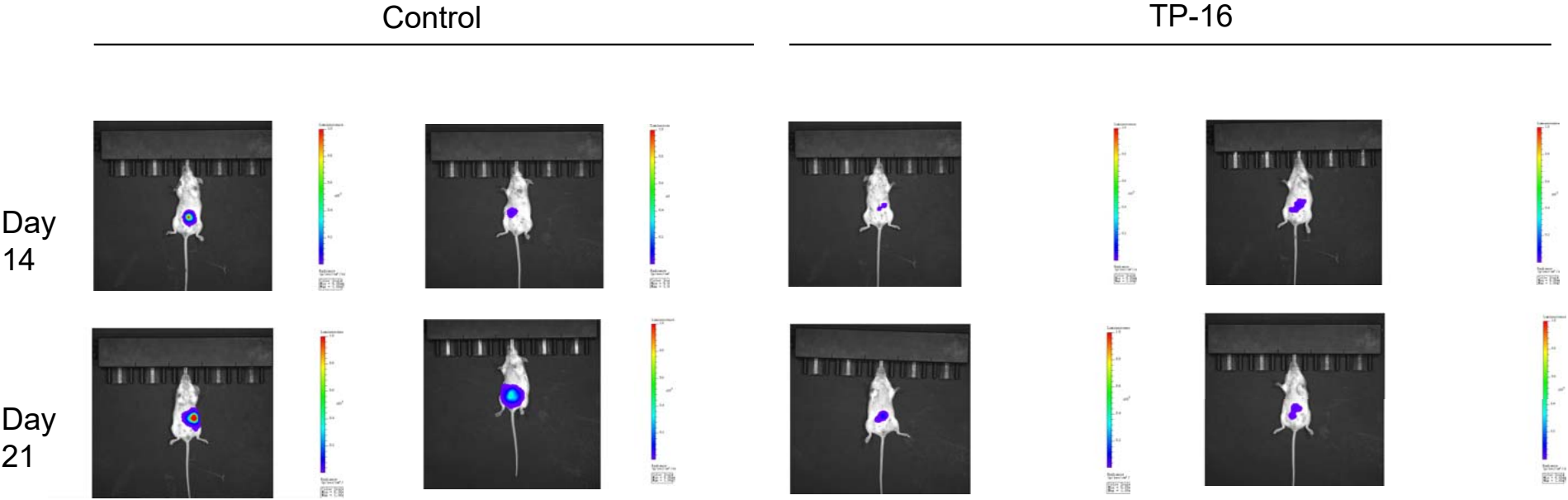

Supplement: Supplementary file 5 — Source Data for Figure 3 [file EMMM-13-e12798-s003.pdf]

Figure 6C

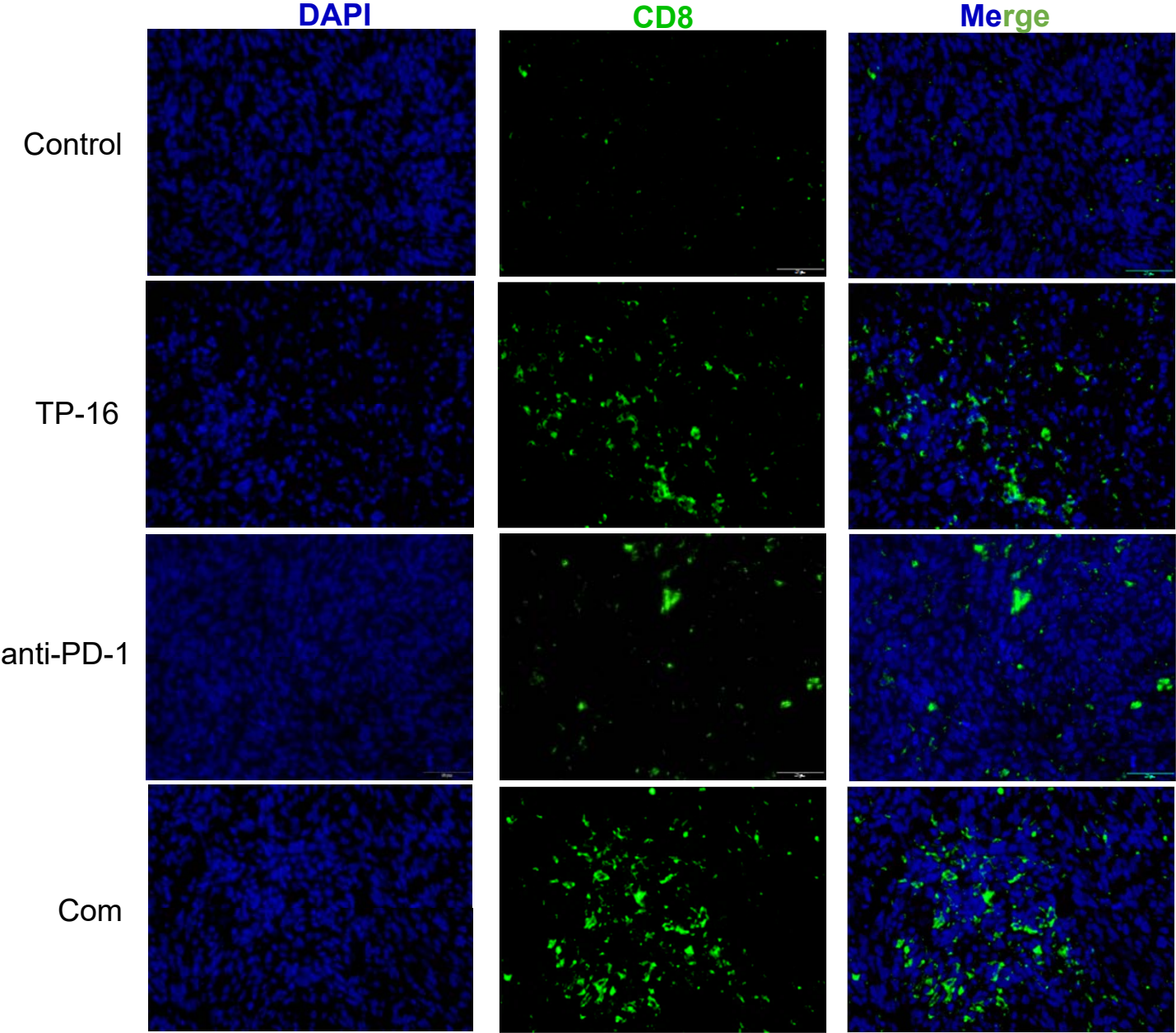

**Figure 6D**

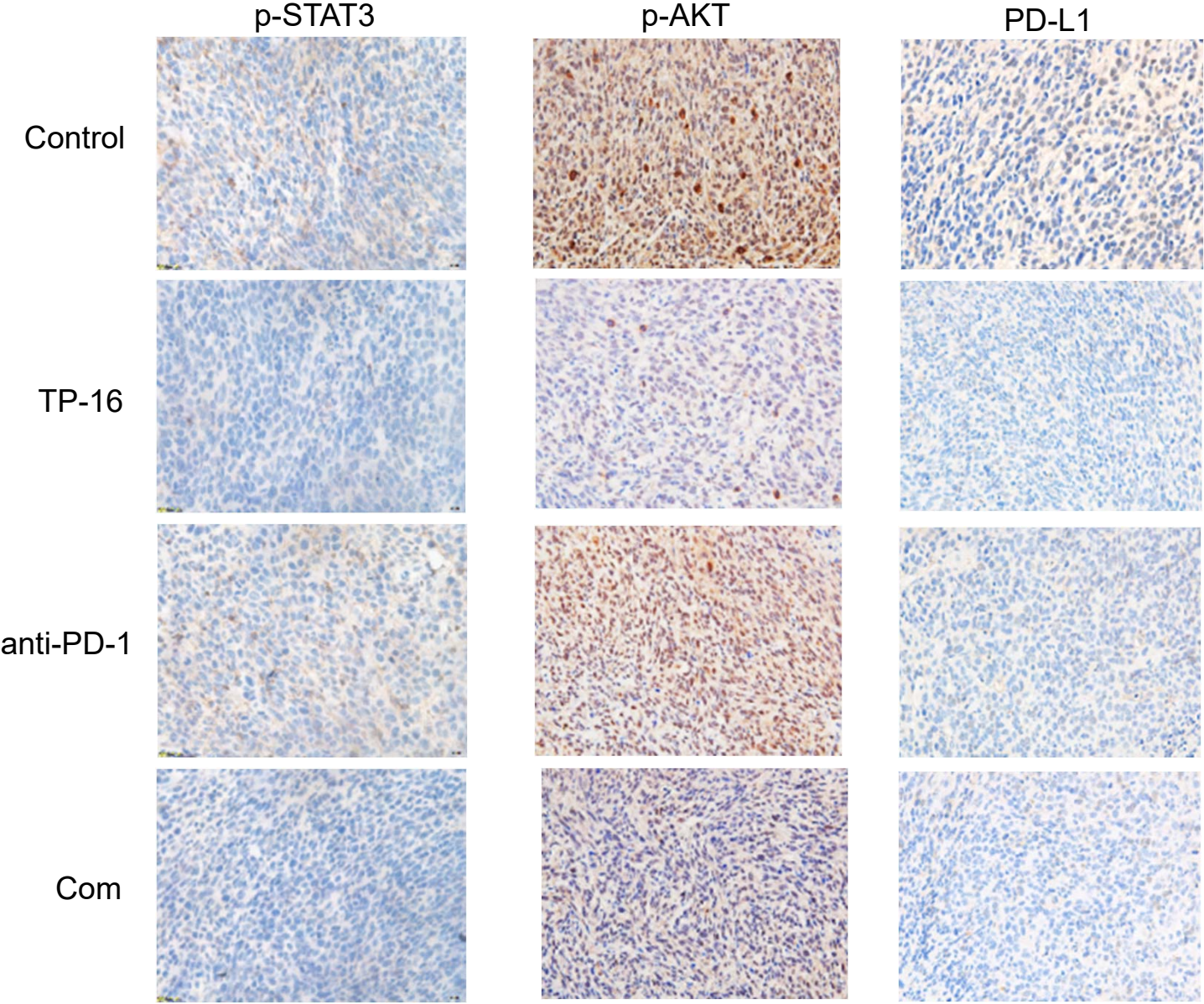

Supplement: Supplementary file 6 — Source Data for Figure 6 [file EMMM-13-e12798-s004.pdf]

Figure 7E

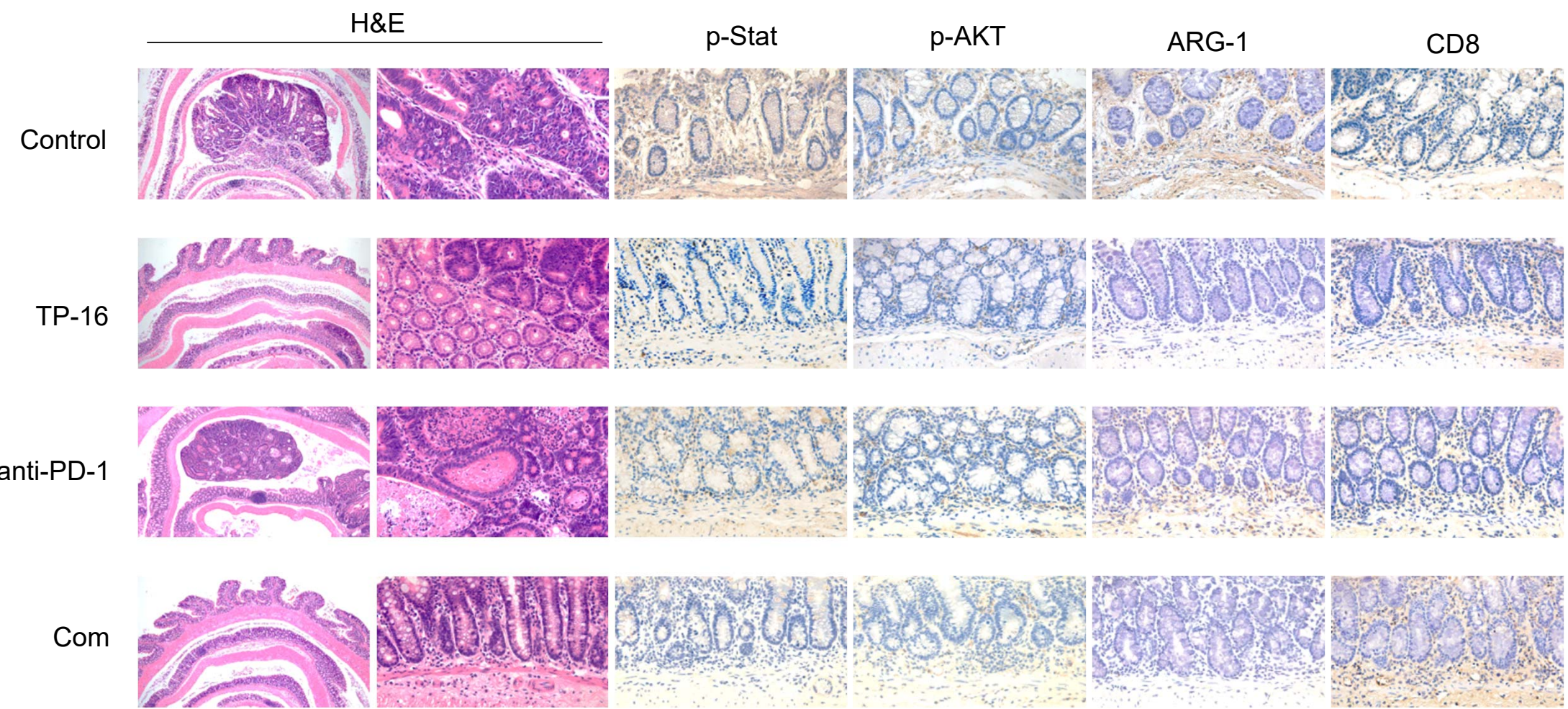

Supplement: Supplementary file 7 — Source Data for Figure 7 [file EMMM-13-e12798-s005.pdf]
