# Supplementary material for: Reprogramming immunosuppressive myeloid cells facilitates immunotherapy for colorectal cancer
Source: EMBO Mol Med. 2020 Dec 7;13(1):e12798. doi: 10.15252/emmm.202012798 (PMC7799360; doi:10.15252/emmm.202012798)
Supplement: Supplementary file 3 — Source Data for Expanded View and Appendix [file EMMM-13-e12798-s006.zip › EMM-2020-12798-V4-EV Figure Source Data.zip!/EMM-2020-12798-V4-Figure_EV4_source_data-sd.pdf]

## Figure EV4D

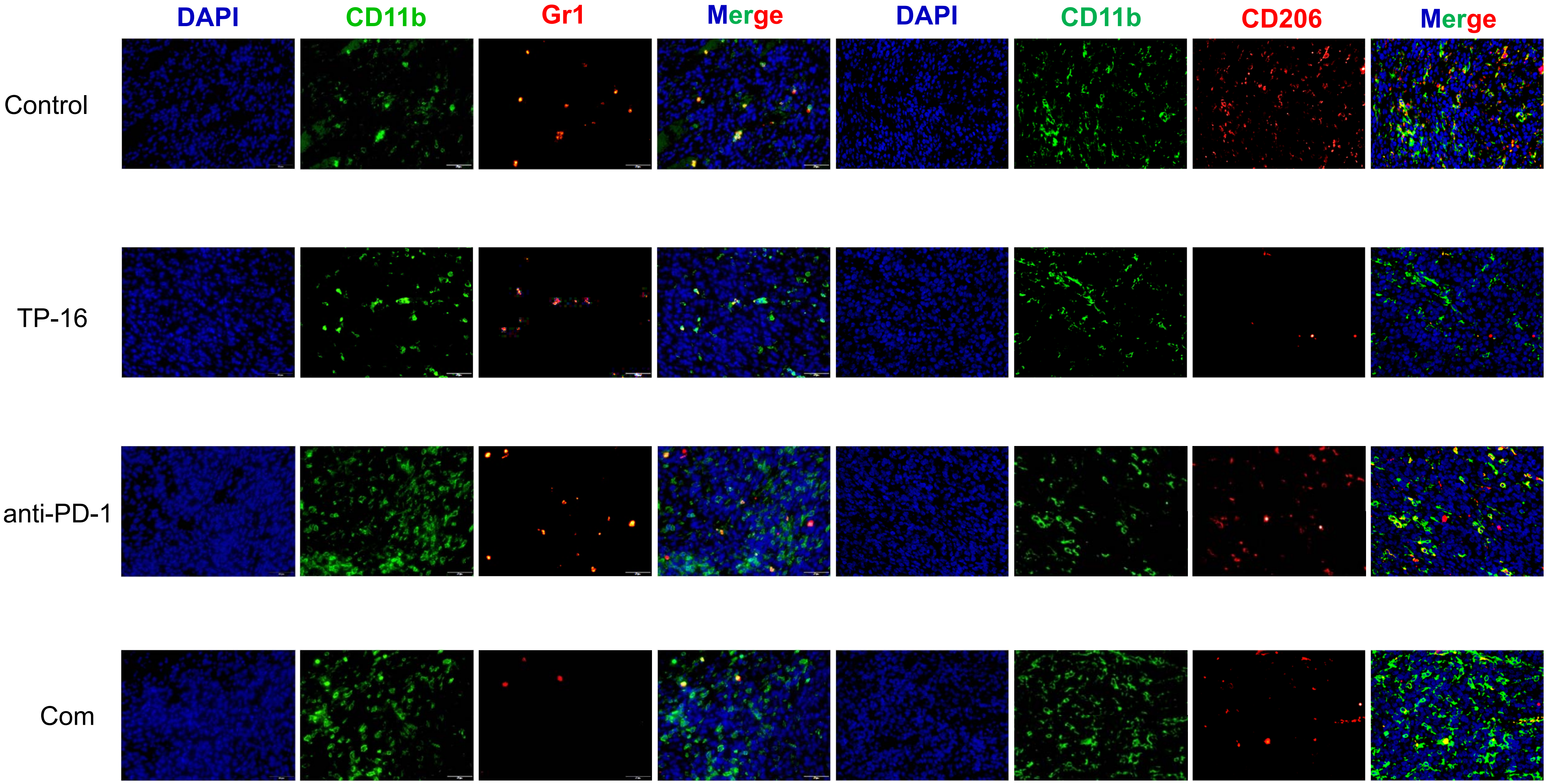

DAPI
CD11b
Gr1
Merge
DAPI
CD11b
CD206
Merge
Control
TP-16
anti-PD-1
Com
